# *In Vitro* Inflammation Inhibition Model Based on Semi-Continuous Toll-Like Receptor Biosensing

**DOI:** 10.1371/journal.pone.0105212

**Published:** 2014-08-19

**Authors:** Jin-Woo Jeon, Un-Hwan Ha, Se-Hwan Paek

**Affiliations:** 1 Department of Bio-Microsystem Technology, Korea University, Anam-dong, Seongbuk-Gu, Seoul, Korea; 2 Department of Biotechnology and Bioinformatics, Korea University, Sejong-ro, Sejong, Korea; University of California Merced, United States of America

## Abstract

A chemical inhibition model of inflammation is proposed by semi-continuous monitoring the density of toll-like receptor 1 (TLR1) expressed on mammalian cells following bacterial infection to investigate an *in vivo*-mimicked drug screening system. The inflammation was induced by adding bacterial lysate (e.g., *Pseudomonas aeruginosa*) to a mammalian cell culture (e.g., A549 cell line). The TLR1 density on the same cells was immunochemically monitored up to three cycles under optimized cyclic bacterial stimulation-and-restoration conditions. The assay was carried out by adopting a cell-compatible immunoanalytical procedure and signal generation method. Signal intensity relative to the background control obtained without stimulation was employed to plot the standard curve for inflammation. To suppress the inflammatory response, sodium salicylate, which inhibits nuclear factor-κB activity, was used to prepare the standard curve for anti-inflammation. Such measurement of differential TLR densities was used as a biosensing approach discriminating the anti-inflammatory substance from the non-effector, which was simulated by using caffeic acid phenethyl ester and acetaminophen as the two components, respectively. As the same cells exposed to repetitive bacterial stimulation were semi-continuously monitored, the efficacy and toxicity of the inhibitors may further be determined regarding persistency against time. Therefore, this semi-continuous biosensing model could be appropriate as a substitute for animal-based experimentation during drug screening prior to pre-clinical tests.

## Introduction

Inflammation is the body's immediate response to damage to its tissues and cells by harmful stimuli, such as pathogens, damaged cells, or irritants [1]. Acute inflammation lasting a short-term period usually not only cures the body from wounds and infections, but also enables the organism to survive via progressive destruction of tissue. However, inflammation is also accompanied by heat, vasodilation, constriction or dilation in vascular smooth muscle cells and eventually physical pain. Chronic inflammation is a prolonged, dysregulated, and maladaptive response, causing various diseases [2], such as hay fever, periodontitis, atherosclerosis, rheumatoid arthritis, and even cancer (e.g., gallbladder carcinoma) [3]. The processes by which acute inflammation is initiated and develops are well defined, but much less is known about the causes of chronic inflammation and the associated molecular and cellular pathways [2]. Therefore, although inflammation is normally closely regulated by the body, it should be monitored and controlled by medication to alleviate symptoms and to prevent inflammation-related diseases from turning into a serious illness [3].

Development of therapeutic drugs including inflammation inhibitory substances relies heavily on *in vivo* animal models to investigate their efficacies and toxicities [4]. Animal models are numerous using various species (e.g., guinea pig, rat, and mouse) for preclinical evaluation of, for examples, anti-inflammatory activity [5]–[7] and anti-cancer drugs [8], [9]. Investigations of insecticidal toxicity after the chemical treatment are also carried out with animal to observe immunosuppression via cytokine measurement [10]. Genetic knockouts or transgenic overexpression animal models are used to collect data on susceptibility to a disease or data on chronic response to stimulation, respectively [11]–[13]. However, such *in vivo* testing can result in a high cost for pharmaceuticals and, more importantly, problems related to ethics [14]. Because animal use is due to a lack of proper *in vitro* model systems that can replace costly and time-consuming animal studies, different cell culture platforms have attracted attention [14]. Relatively small-sized animal model is also emerging for biomedical and environmental toxicological testing using a saprophytic nematode species such as *Caenorhabditis elegans* [15]–[17].

**Figure 1:**
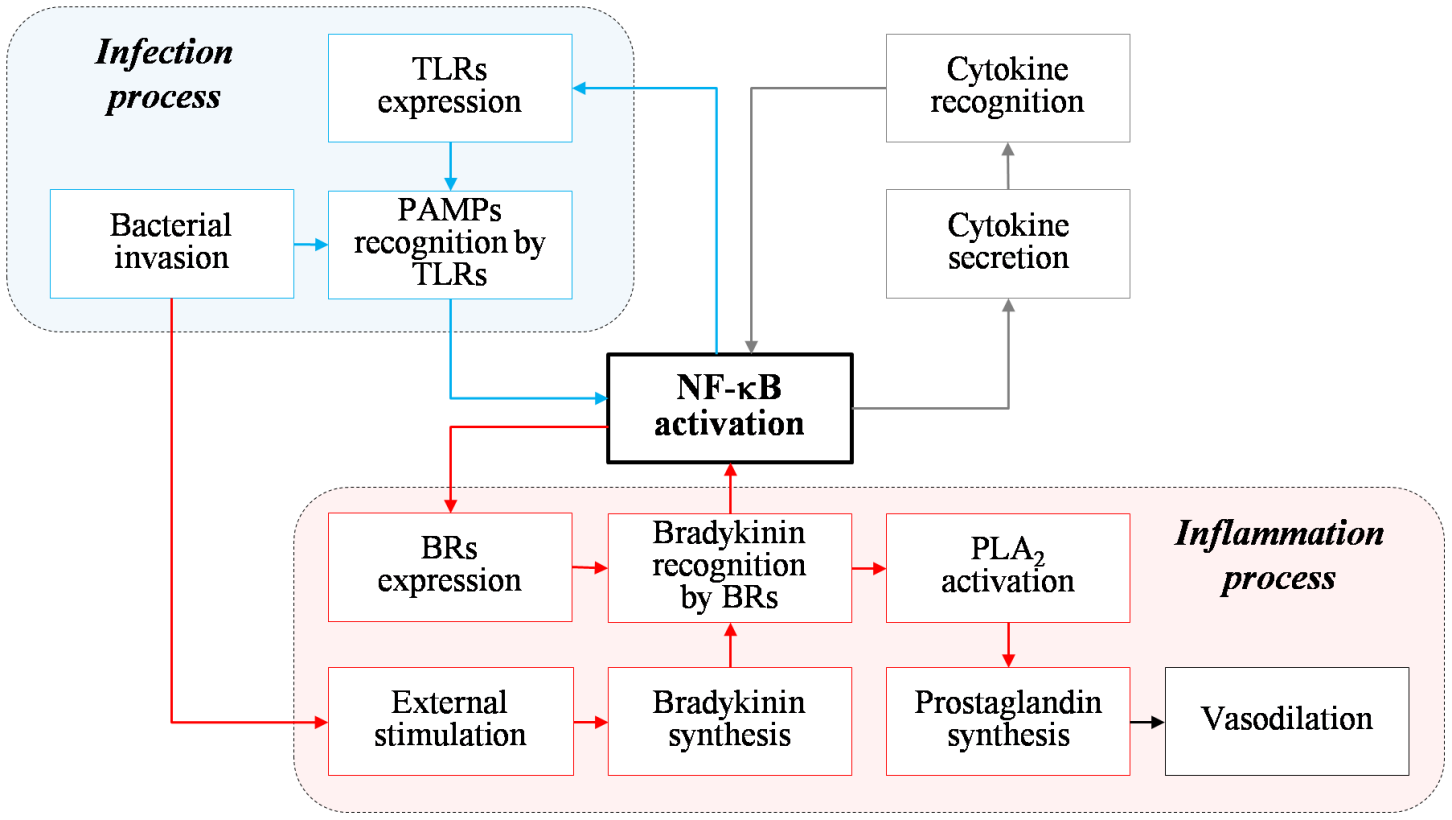
Mechanism of inflammatory response via NF-κB activation against bacterial invasion. Inflammation proceeds mainly via two different routes, which are initiated by TLRs-PAMPs binding (Infection process) or bradykinin-BR interactions (Inflammation process). The former invokes inflammation by producing cytokines as a result of the response. NF-κB activation also increases the densities of other mediators such as TLRs and BRs.

In the case of pathogenic infection, inflammatory responses in host cells are induced through recognition of the conserved regions of pathogenic microorganisms, i.e., pathogen-associated molecular patterns (PAMPs), by toll-like receptors (TLRs) [18]. Each TLR reacts with different types of pathogenic ligands (e.g., di-acylated lipopeptide, tri-acylated lipopeptide, and lipopolysaccharide). Binding initiates the inflammatory response in host cells by activating signal transduction pathways mediated by transcription factors, i.e., nuclear factor-κB (NF-κB; Figure 1, “Infection process”; [18]). This transcription process then produces cytokines, which mediate cell-to-cell communication to propagate the infection signal. Such cellular responses also include expression of TLRs, G-protein coupled receptors, and bradykinin receptors (BRs) on cell surfaces [19].

Bacterial invasion can also stimulate activation of the kinin-kallikrein system and produce bradykinin, another inflammatory mediator, by interacting with BRs (Figure 1, “Inflammation process”; [20]). Such interactions also activate the NF-κB pathway to produce BRs and prostaglandins. These are precursory reactions of the inflammatory response, which eventually lead to vasodilation. The inflammatory response can also be induced by other external stimuli such as burns or physical trauma by activating the same NF-κB pathway [1]. Activation of the kinin-kallikrein system on damaged cells is poorly understood [21]. Such a cellular response also increases the levels of receptors as inflammatory mediators.

The progression of inflammation and effects of medications can be monitored by examining either the changes in inflammatory mediator levels or NF-κB activity [22]. First, the mediator concentrations of bradykinin and prostaglandin can be determined either by measuring the mRNA expression levels of receptors via the polymerase chain reaction [23] or by competitive binding assays using a radioisotope as a tracer [24]. However, these methods require either cell disruption to extract genetic molecules, or collection of the binding complexes present on the cellular membrane. Enzyme-linked immunosorbent assays are also available for the analytes in plasma sample [25], still the sensitivity is limited for bradykinin in normal plasma [26] and the result was not reliable for prostaglandin due to the instability in real sample [27], [28]. Nitric oxide, another inflammatory mediator, can also be traced for the same purpose [29]; however, this has low specificity. Alternatively, assays for NF-κB activity have been developed [30], which also require cell lysis to extract NF-κB or to enhance permeability of an antibody tracer across the cellular membrane. Another way of detecting NF-κB activity is the indirect approach of immuno-analyses using various markers that indicate activity, such as BRs, TLRs, and cytokines [31]–[33]. As these are present on the cellular membrane surfaces or secreted, they can be monitored via immunoassays using antibodies specific to each target, and such methods do not require destruction of the cells for activity monitoring.

In this study, we propose to use TLRs as indicators of the cellular response to infection as they are readily measured by employing immunoassay techniques. Using TLRs as the markers, we have previously investigated a hypersensitive biosensing system employing animal cells for externally invading microorganisms [34]. We further developed a semi-continuous monitoring technique to record the responses of the same cells to repetitive infections [35]. In the latter study, experimental conditions were established to semi-continuously monitor the inflammation level in the same cells by adding novel features to conventional methodology, including TLR regulation switching, cell fixation-free analysis of the marker, and signal generation with minimal cell damage [35]. These technologies may not enable us to monitor only the activation status of NF-κB by infection, but also the regulation by an anti-inflammatory drug (e.g., aspirin [36]). Such inhibition blocks recycling of NF-κB, as this transcription factor is normally coupled with Iκβα in the cytoplasm to maintain its inactive state [36]. Therefore, TLRs are not further produced while PAMP-bound receptors are internalized via endocytosis, resulting in a decrease in receptor density. The cellular responses regarding TLRs to repetitive infections can be obtained in reproducible manner [35], which may allow us to readily recognize the NF-κB inhibition via pattern analysis.

Semi-continuous biosensing technology was applied to construct an experimental model suitable for anti-inflammatory substance screening, which provides further information besides efficacy. Sodium salicylate, which inhibits NF-κB activity [22], [36], was used to prepare a TLR suppression chart. This inhibition process may be similar to the *in vivo* mechanism in which an anti-inflammatory drug (e.g., aspirin) was used to inhibit NF-κB activation. Screening was then simulated by additionally employing positive or negative substances, regarding the inhibition of activity, respectively. As we adopted a semi-continuous biosensing technique to quantify TLR density, the efficacy and toxicity of the inhibitors on the cells can be determined regarding persistency against time. Such testing is currently carried out through experimentation using animals and, therefore, a substitute methodology is needed [14], [37]. In this study, an experimental model was established to inhibit TLR1 expression on a human adenocarcinoma cell line such as A549 in response to bacterial stimulation by *Pseudomonas aeruginosa*, causing pulmonary inflammation, in a lysate form. The bacterial preparation was used to precisely control the stimulation dose and consequently to obtain a reproducible cellular response pattern in a semi-continuous manner. A typically renowned human cell line, HeLa, was additionally sought to use it for simultaneously testing for the inflammation status, and a third murine cell line, RAW264.7, was also invited as animal species control for comparison. As the used cells were all immortalized cell line, the anti-inflammatory effects presented in this study might be different from those obtained *in vivo*.

## Materials and Methods

### Materials

All reagents used were of analytical grade and are specified in Text S1.

### Preparation of Bacterial Lysate and Mammalian Cells

The *P. aeruginosa* bacterial lysate (PAK strain) was prepared by sonication as described previously [38]. Briefly, the bacterium was grown in Luria–Bertani broth, and the cell pellets were suspended in a phosphate buffer containing NaCl (PBS), boiled, and sonicated. The supernatant was diluted to a protein concentration of 0.1 mg/mL with PBS and stored at −80°C after being snap-frozen in aliquots.

The A549 mammalian cell line was maintained in RPMI1640 culture medium, supplemented with fetal bovine serum (FBS) and antibiotics, according to standard protocols [39]. For storage, the cells were diluted to 3×10^5^ cells/mL in culture medium containing 5% FBS and 10% dimethyl sulfoxide, and snap-frozen in liquid nitrogen. Experimental details not mentioned in this section are described in Text S1.

### Analytical Procedures for TLR Induced by Bacterial Stimulation

The mammalian cells were pre-cultivated prior to bacterial stimulation by suspending them in supplemented culture medium to stably attach the cells to the bottom of a 96-well culture plate. After washing, serum-free medium was added to the wells and incubated for 2 h for starvation. The pre-cultivated cell culture was stimulated with the bacterial lysate for 2 h. TLR1 was quantified by adding an antibody specific to the receptor and sequentially reacting with anti-rabbit IgG labeled with HRP. An HRP substrate solution containing TMB was then added to the well and allowed to react to generate the color signal, and the color was measured at an absorbance of 450 nm after adding sulfuric acid. Mean and standard deviation values were used to plot the receptor expression level with variation against the target variable by using the Excel program from Microsoft. Data are presented as mean ± standard deviation (SD). Statistical analyses were performed using one-way analysis of variance followed by Tukey's post hoc multiple range tests [40] using the SPSS software package for Windows (SPSS Inc.; Chicago, IL, USA). A P<0.05 was considered significant.

The TLR1 expressed on the cells surface was also measured using a HRP chemiluminometric substrate to minimize cellular damage. The luminescent signal was measured using a cooled charge-coupled device (CCD) camera under dark conditions, and the captured images were digitized. The optical densities were integrated and used to plot the standard curve using the Excel program, and statistical testing were also carried out as mentioned. Experimental details are presented in Text S1.

### Suppression of Cell Receptor Upregulation Using a Chemical Inhibitor

The inhibitory conditions were optimized via TLR-based colorimetric detection by selecting sodium salicylate as the NF-κB pathway inhibitor. A549 cells were first attached to the bottom of a plate and treated with the inhibitor by co-incubation with the bacterial lysate or a pre-incubation, in which the inhibitor was added first at the time of starvation and then stimulated with the lysate in the next step. The rest of the procedure remained the same as indicated above except for the negative control without inhibitor. The same protocol was applied for caffeic acid phenethyl ester (CAPE) and acetaminophen inhibitor testing. Detailed protocols are presented in Text S1.

### Semi-continuous Biosensing Model for Anti-inflammation

The cellular response to repetitive bacterial stimulations was monitored by immunochemically measuring the TLR1 level according to stimulation-and-restoration cycle. The TLR1 background luminescent signal level was measured in pre-cultivated cells, and the cells were washed immediately. The cell culture was starved in the first cycle, stimulated with the bacterial lysate, and the TLR1 density was determined via immunoassay. The same culture was subsequently restored in serum-containing medium. The same procedure was repeated for the next cycles. The whole process was negatively controlled by not adding stimulus agent, which was used to yield the signal-to-background ratio against time.

Sodium salicylate was incubated either with the bacterial lysate at the same time or by a pre-incubation at the starvation step to construct the inhibitory inflammation model. Cellular responses were cyclically monitored twice, and the patterns were compared with that of the control without inhibitor.

CAPE and acetaminophen were used as positive and negative regulators to simulate anti-inflammatory substance screening. Prior to testing, two standard curves were prepared for inflammation against repetitive bacterial stimulation and for inhibition in the presence of sodium salicylate. Each process was negatively controlled without stimulation such that the signal-to-background ratio could be calculated to plot the standard curves against time. The two candidates were then tested by serially adding each to the cell culture according to the scheme of semi-continuous biosensing as described.

The same procedure as that used for inhibition testing was used to estimate drug effect persistency for sodium salicylate, except we omitted the chemical treatment in one of the two cycles after adding it in the first cycle. As described above, two standard curves were prepared and used to compare the persistency testing results. Experimental procedures and conditions are described in detail in the Text S1.

## Results and Discussion

The goal of this study was to propose an experimental model suitable for anti-inflammatory substance screening, which has been conventionally carried out using animals. To investigate the new model, we established the biological concept, described earlier, for the expression of TLRs as a biomarker indicating the status of inflammation by bacterial stimulation and also showing its regulation. The concept was then visualized to an experimental model of inflammation inhibition using a well known drug substance, for which a repetitive TLR monitoring technique for the same cells, i.e., semi-continuous biosensing, was employed. The model was simulated for inhibitor screening and finally characterized toward the performances. These results are presented in the sections below.

### Establishing an Experimental Model for Inflammation Inhibition Monitoring

We selected sodium salicylate as the test chemical, as it is salicylate similar to aspirin [22]. Sodium salicylate is a well known inhibitor of the NF-κB pathway initiating the inflammatory response. Although NF-κB is normally coupled with Iκβα, bacterial infection induces a signal to activate Iκβ kinase, which phosphorylates and then ubiquitinates Iκβα and consequently removes it from the complex. The free NF-κB becomes active, moves into the nucleus, and combines with DNA for transcription to produce mediators related to inflammation. During such an inflammatory process, sodium salicylate produces an anti-inflammatory effect by competitively binding with ATP, which is required as a co-substrate of Iκβ kinase [22].

#### TLR expression by bacterial stimulation

We initially selected three Gram-negative bacteria, *P. aeruginosa*, *Shigella sonnei* (*S. sonnei*), and *Vibrio haemolyticus* (*V. haemolyticus*), prepared in lysate form. They were then added respectively on different host cells: two human cell lines, A549 and HeLa, and a murine cell line, RAW264.7. Among the TLRs present on the cell membrane surfaces, TLR1, TLR2, and TLR4 were used as the sensing elements for PAMPs. The host cells were stimulated with each bacterium at a concentration (0.001 mg/mL lysate proteins corresponding to 1×10^5^ cells/mL for *P. aeruginosa*, 5×10^4^ cells/mL for *S. sonnei*, and 2×10^5^ cells/mL for *V. parahaemolyticus*). TLR expression levels after bacterial stimulation were detected using the respective anti-TLR antibodies, which indicate the degree of interaction between the host cells and stimulus agents (Figure S1). In these experiments, the cell lines responded to the bacterial stimulation in a similar fashion although the TLR levels were relatively different among the host cells. In the case of *P. aeruginosa* or *V. haemolyticus* stimulation, only the TLR1 response was significant compared to each background (None) for all cell line according to statistic analysis. In contrast, for *S. sonnei* stimulation, expression of all tested TLRs was higher than the backgrounds in the order of TLR4>TLR2>TLR1. Accordingly, the expression level of each marker could be determined by the PAMPs present on the stimulus agent.

As human cells (e.g., A549 and HeLa) will be needed to eventually utilize this model for human drug investigation, A549 offering a relatively low background was eventually selected as a host in this study. A comparative study utilizing a chemical inhibition model of inflammation was then conducted by devising two distinct models: TLR1 response to *P. aeruginosa* (Model A) and TLR4 response to *S. sonnei* (Model B) on the same cells (A549). These models displayed similar expression levels for the same lysate titer of each bacterium (refer to Figure S1A).

#### Suppressing TLR up-regulation

**Figure 2:**
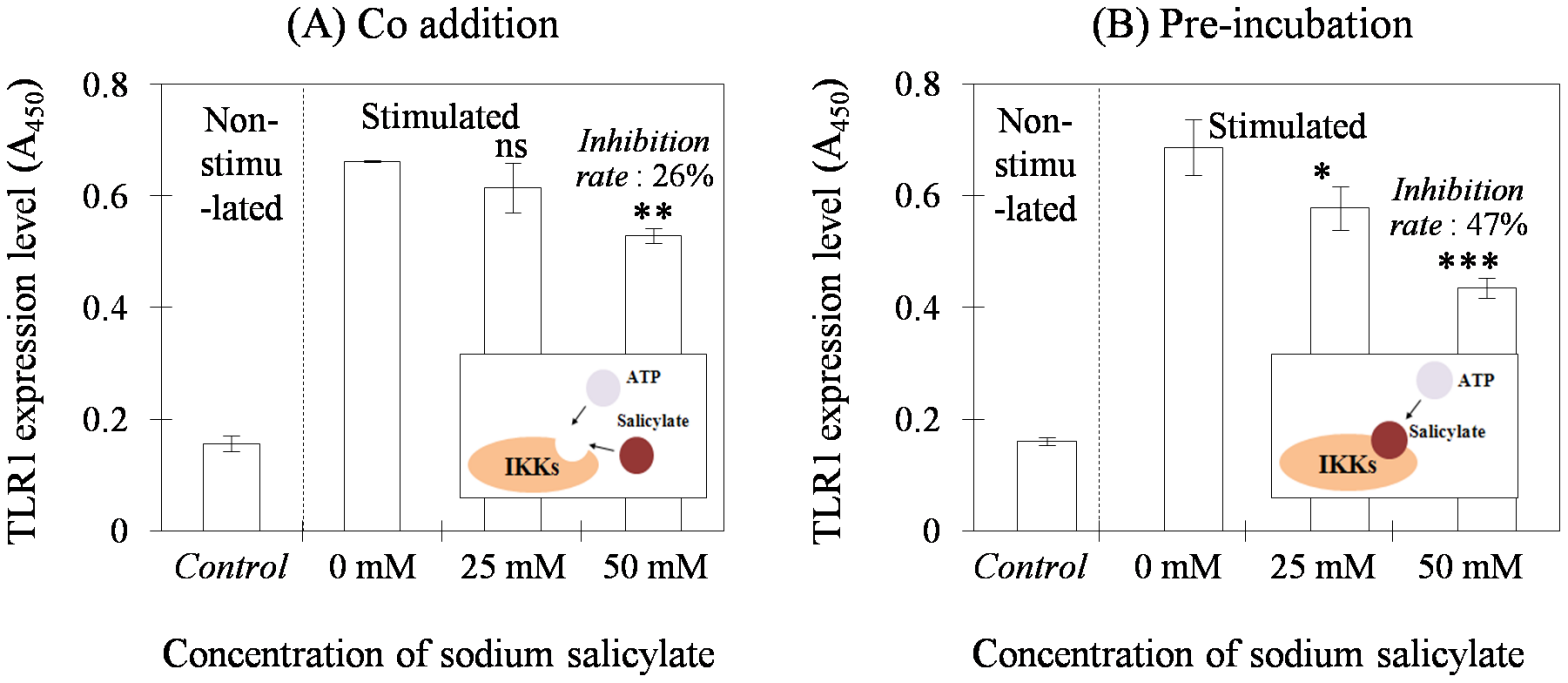
Testing the effective dose and treatment timing, relative to bacterial stimulation, of sodium salicylate as an anti-inflammatory substance. Experimental data were obtained by measuring the signals in colorimetric mode, which represented the TLR1 density expressed on the cell surfaces (A549). When stimulated with the *P. aeruginosa* lysate, expression inhibited by sodium salicylate was most effective at a dose of 50 mM and with pre-incubation of the drug, i.e., pre-incubation in B. Data are shown in mean ± SD (n = 3) and comparisons to the control (i.e., stimulated cells without anti-inflammatory substance treatment) are marked as *** very highly significant (P<0.001), ** highly significant (P<0.01), or * significant (P<0.05). No significance (ns) was indicated otherwise.

Sodium salicylate treatment conditions were first optimized by using Model A regarding dose and timing of addition to the cell culture (Figure 2). High doses such as >100 mM were harmful to the cells (data not shown). As a result, the level of activated TLR1 (0 mM sodium salicylate; Figure 2A) was inhibited directly proportional to the drug dose used when the drug was added with the bacterial lysate. The maximum inhibition percentage was about 26% when the cells were treated with 50 mM sodium salicylate. For comparison, sodium salicylate was first added at the starvation stage and the bacterial stimulation was subsequently conducted (Figure 2B). Such an approach further increased the inhibition percentage up to 47%. Such an enhanced anti-inflammatory effect may have resulted from the pre-incubation of the cells, which favored sodium salicylate in the competitive reactions between the drug and ATP with Iκβ kinase. The effect was further checked by testing inhibition of BR, another inflammatory mediator, at the molecular level (Figure S2).

The significant inhibitory effect of sodium salicylate was consistent with the result shown in an earlier report and may have occurred due to slow dissociation of the substance from the Iκβ kinase complex [36]. In a previous study, aspirin (acetyl salicylate) and sodium salicylate were used as enzyme inhibitors, and pre-incubation of each drug also revealed a greater inhibitory effect than that in a simultaneous addition. Here, a purified form of the enzyme was employed so strong inhibition was observed with a lower concentration than that used in this experiment. The cell culture system in the present study required mass transport across the cellular membrane but hydrolysis of sodium salicylate may have occurred during incubation in aqueous medium. In an animal model, sodium salicylate shows lower suppression potency during prostaglandin synthesis than that of aspirin [41]. It should be noted that when the anti-inflammation agent was added at >100 mM, the cells were damaged and detached from the solid surfaces by apoptosis. The cell damage may have been caused by DNA fragmentation and proteolytic cleavage of poly(adenosine 5′-diphosphate-ribose) polymerase, depending on the drug concentration [42].

#### Semi-continuous approach for the inhibition model

Because the up-regulated TLR level due to bacterial stimulation can be returned to baseline via receptor regulation switching, the semi-continuous biosensing concept was applied to test of the anti-inflammatory drug effect. Over-expressed TLRs are down-regulated through either receptor-mediated endocytosis or clathrin-dependent endocytosis [43]. Such a regulatory mechanism has been imitated in cell culture, and repeated bacterial stimulations could be monitored by recycling the same cells without fixation [35]. This semi-continuous biosensing approach would be valuable as a potential animal model substitute, particularly for drug toxicity testing.

**Figure 3:**
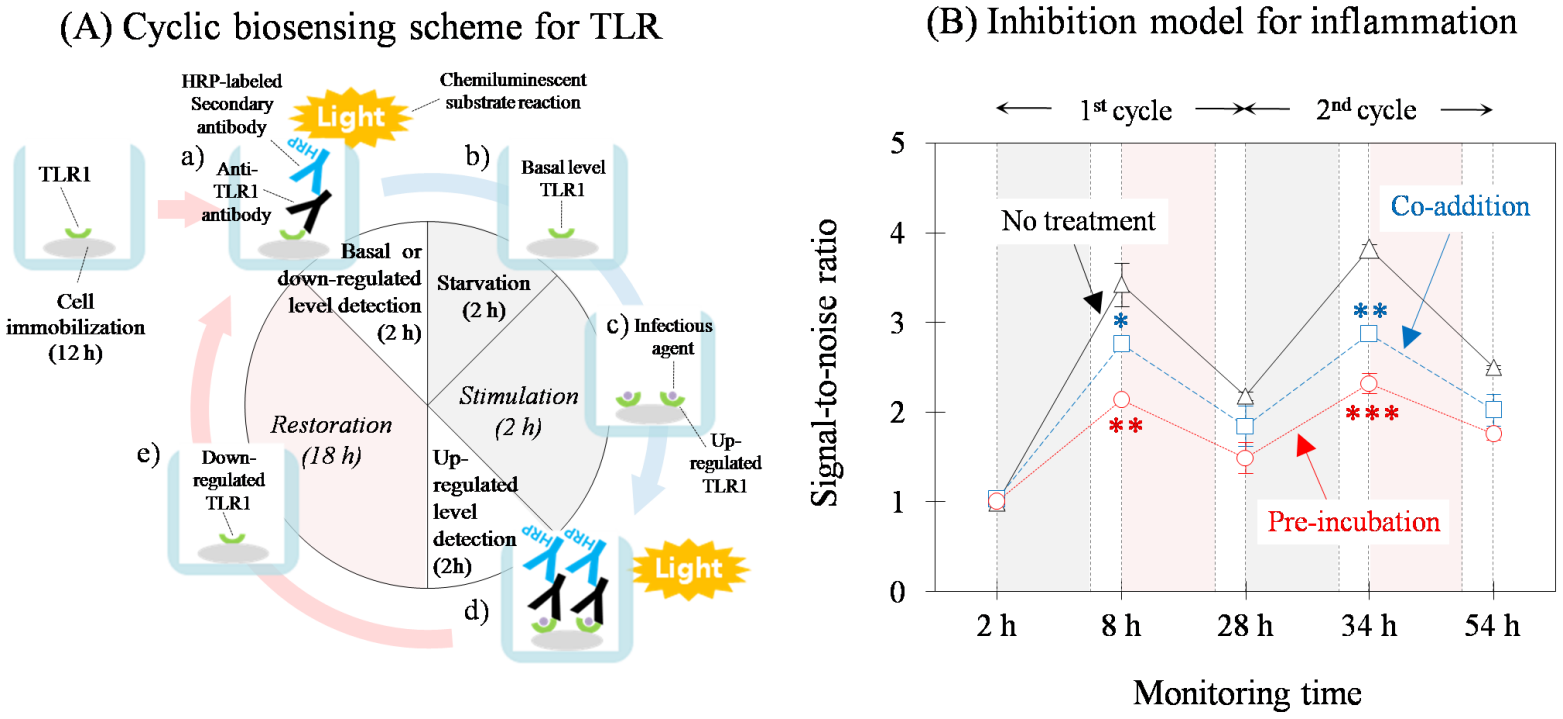
Application of the semi-continuous analytical approach for TLR regulation to test the anti-inflammatory substance effect. Optimal conditions for the TLR regulation scheme were determined (A) and TLR expression was observed in response to two cyclic repeated bacterial stimulations (B, No treatment). Sodium salicylate was the positive inhibitor (see Figure 2) and an effective dose (50 mM) was used either by co-incubation with the stimulus agent (Co-addition) or by pre-incubation (Pre-incubation). Both treatments revealed inhibition of the TLR response to serial stimulations, suggesting a model for anti-inflammatory substance screening. Each measurement was repeated twice under identical conditions and comparisons to the control (No treatment) were marked as stated in Figure 2 legend.

The analysis required us to control a considerable number of factors to vary the expression yield of TLRs such as host cell line, stimulus agent type, target receptor, cell culture conditions, and stimulation timing, which were determined in a previous study [35]. The initial time for each cyclic response using Model A was as follows except for pre-cultivation during cell attachment for 12 h (Figure 3A): a) basal or down-regulated detection level for 2 h, b) starvation for 2 h, c) stimulation for 2 h under starvation conditions, d) up-regulated level detection for 2 h, and restoration for 18 h in serum-containing medium. This stimulation-and-restoration cycle for the same cell culture was repeated twice to semi-continuously observe the cellular response to TLR expression (Figure S3B) using a cooled CCD-based luminescent biosensing system developed by us (Figure S3A; [35]). TLR density of a non-stimulated cell culture was also measured as a control. The relative value of the two densities at each stage, which is the signal-to-noise ratio, explicitly showed an up-and-down response pattern twice, although both the maximum and minimum values in each cycle increased slightly (Figure 3B, No treatment). This result adequately demonstrated semi-continuous monitoring of a mammalian cell response to external stimulation.

The anti-inflammatory effect of sodium salicylate was further tested by following the TLR biosensing scheme using the optimal inhibition conditions determined above. The test drug (50 mM) was added either simultaneously with the bacterial lysate or sequentially into the cell culture, and the results were compared with those of the control without treatment (Figure 3B; refer to Figure S3C and S3D for TLR data). During two cyclic responses of the cell culture to repetitive bacterial stimulations, the co-addition in each cycle was reproducibly effective for suppressing bacterial inflammation (Figure 3B, Co-addition). Pre-incubation showed an enhanced anti-inflammatory effect, as expected (Figure 3B, Pre-incubation). Similarly, the same pattern of inhibited inflammation was reproducibly shown with Model B using a different pair of stimulus agent and cellular receptor (Figure S4). The results from the collective inflammation study may support the measurement of differential TLR densities as a biosensing approach with possible discriminatory ability between the anti-inflammatory substance and non-effector. It is conceivable from these results that the semi-continuous responses to repetitive stimulations would certainly offer accumulated information about drug efficacy and toxicity using the same cells. This will be further demonstrated below by introducing another anti-inflammatory substance and by extending the cyclic response frequency.

### Simulation of Anti-inflammatory Substance Screening

**Figure 4:**
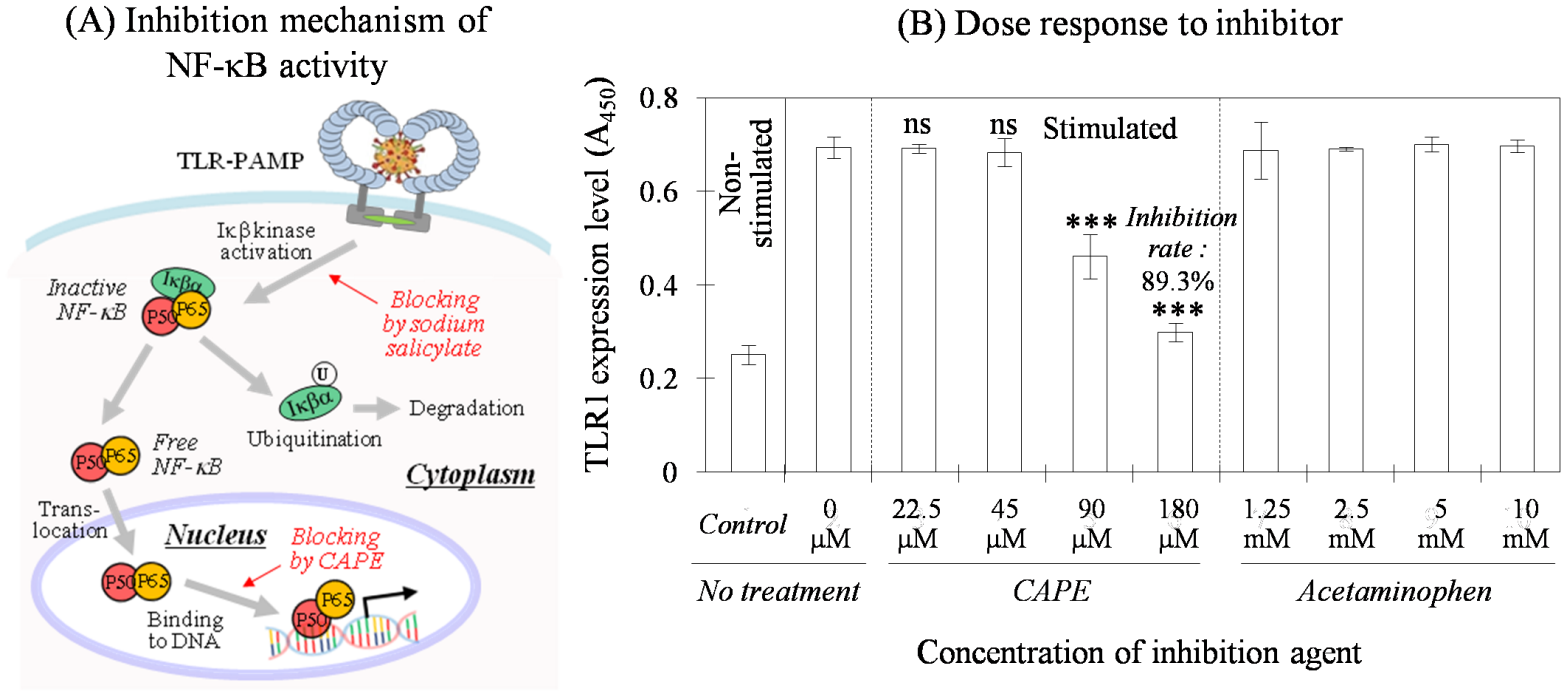
Schematic representation of NF-κB inhibition by CAPE and dose response of the cells to the inhibitor. The inhibition site in the NF-κB activation pathway for CAPE is very different from that of sodium salicylate (A). CAPE effectively suppressed up-regulation of TLR1 density when stimulated with the bacterial lysate, and the degree was proportional to the concentration used (B). This compared with the results of no response to acetaminophen as the negative control. Data were expressed as mean ± SD (n = 4) and each significance was also indicated.

The semi-continuous biosensing model for anti-inflammation was examined to determine whether it could detect a substance showing a similar effect on NF-κB regulation. To this end, we used CAPE and acetaminophen, each of which expresses a positive or no observed effect [22], [44]. CAPE is a substance extracted from beehives that inhibits the NF-κB activation pathway in a mechanism completely different from that of sodium salicylate (Figure 4A). The free form of NF-κB is produced in the cytoplasm by eliminating Iκβα from the complex and is translocated to the nucleus [22]. Here, CAPE modifies a sulfhydryl group of the p65 subunit of the NF-κB molecule, which is the DNA binding site, and represses inflammatory activity [44].

In contrast, acetaminophen affects NF-κB activity differently [45]. Although the action mechanism has not been completely understood to date, its peripheral anti-inflammatory activity is poor and usually limited by several factors. Acetaminophen weakly inhibits cyclooxygenase (COX) involved in prostaglandin synthesis (refer to Figure 1) and is selective for COX-2 [46]. Therefore, acetaminophen was used a negative control of NF-κB activity in this study.

#### Variable mechanisms of anti-inflammation

Such two substances acting distinctly on the inflammation pathway were tested for their effect on TLR1 expression when cells were stimulated with bacterial lysate (Figure 4). Without chemical treatment, the bacterial stimulation reproducibly increased about 2.8 times the receptor level of host cells compared to that of the non-stimulated cells (Figure 4, No treatment). CAPE was added to the culture to examine the inhibitory effect and TLR1 level decreased compared to that of untreated cells at ≥90 µM, (Figure 4, CAPE). The degree of inhibition was proportional to the CAPE dose in the range used and reached up to 89.3%. In contrast, when acetaminophen was added to cells in a mM dose range, no effect on receptor suppression was found as expected (Figure 4, Acetaminophen). Treatment with such a high amount of acetaminophen did not significantly affect cell growth.

#### Simulating candidate screening

**Figure 5:**
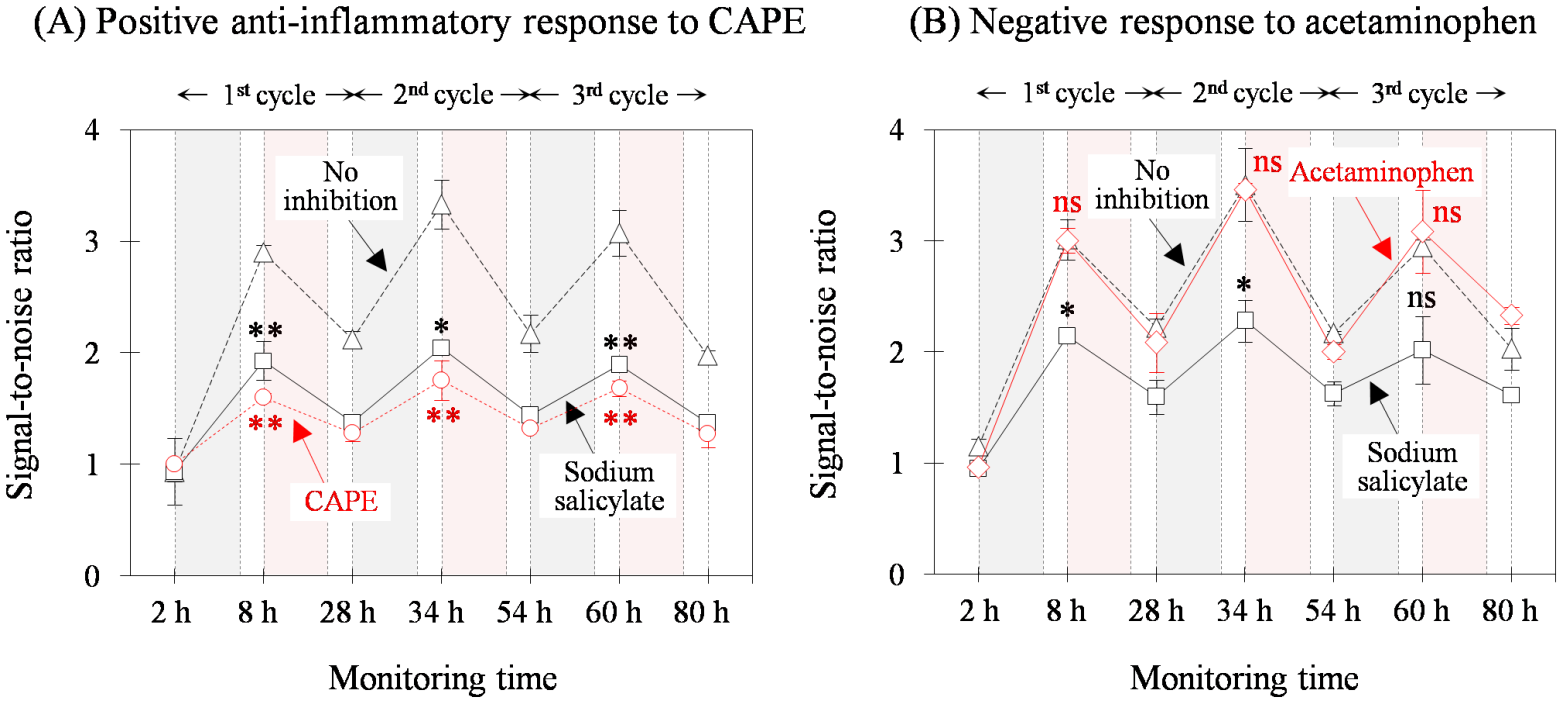
Simulation of anti-inflammatory agent screening based on standard controls in the repetitive inhibition response curve. According to the testing scheme (Figure 3A), the stimulation-and-restoration cycle was repetitively maintained three times without chemical treatment (no inhibition) or separately with sodium salicylate treatment (positive inhibition). When two different substances were employed in the inhibition testing, CAPE (90 µM) showed positive anti-inflammatory response (A) when compared with the two control curves, whereas acetaminophen (10 mM) revealed a negative inhibition response (B). All experiments were carried out in duplicate and comparisons to the control (No inhibition) were marked as mentioned in Figure 2 legend.

We then applied the semi-continuous biosensing technique to test whether these chemicals that inhibited TLR expression could offer additional information such as cell toxicity. The test in this case was extended to three repetitions of the stimulation-and-restoration cycle using the same cells according to the scheme shown in Figure 3A. Prior to testing, negative and positive control inhibition curves were prepared by conducting the cyclic biosensing for cell cultures without added chemicals or with sodium salicylate treatment, respectively (Figure 5). CAPE (90 µM) was then applied and resulted in inhibited TLR1 expression, which was stronger than that obtained with sodium salicylate (Figure 5A). Acetaminophen (10 mM) was tested and showed a cyclic response curve that overlapped with a curve that did not show inhibition (Figure 5B). This result indicated that acetaminophen did not block the NF-κB activation pathway. Although we simulated screening of the anti-inflammatory drug candidates using the substances with known effects, such a strategy could be applied to test unknown samples.

### Characterization for Potential Cytotoxicity and Duration of Inhibition

Semi-continuous monitoring for the cellular response may also offer extra information regarding test substances such as cytotoxic effects and the duration of the anti-inflammatory effect. Most drug screening systems have been developed for high throughput sorting ability to obtain a number of candidate substances [47]. However, such a conventional approach requires separate toxicity tests on cells or animals prior to entering pre-clinical tests. Alternatively, we used our novel technique to identify additional enhanced features.

#### Evaluation of cytotoxicity

The cyclic curves for both test substances were parallel to those of the controls, suggesting that they were not toxic to the cells (Figure 5). This was also confirmed by the morphological observations of the cell. In contrast, cytotoxicity was observed in cultures treated with excess CAPE (180 µM; Figure S5). After pre-incubation of the substance, the stimulation with lysate did not significantly increase the TLR level comparing to that of the non-stimulated control (refer to the bar graphs at 8 h in (B)). This could be caused either by anti-inflammatory effect of the test substance or by its cytoxicity on the cells. Furthermore, the treated cells detached from the solid matrix after the first cycle culture (see Figure S6). This resulted in a substantial increase in TLR levels for both the stimulated and non-stimulated cultures (, see the bar graphs at 28 h in (B) compared to those of the control in (A)). Such cell death caused a distorted signal-to-noise ratio in the corresponding cycle, which was not parallel to the control curves as mentioned (Figure 3B). Such information about cytotoxicity could discriminate the effect by an eye examination for the dose response pattern, which may be unique to the semi-continuous biosensing method. The cytotoxic effect can also be caused in the second or third stage by accumulation if a test drug was repetitively applied to the same cells although it initially revealed anti-inflammatory effect.

#### Estimating effective duration

**Figure 6:**
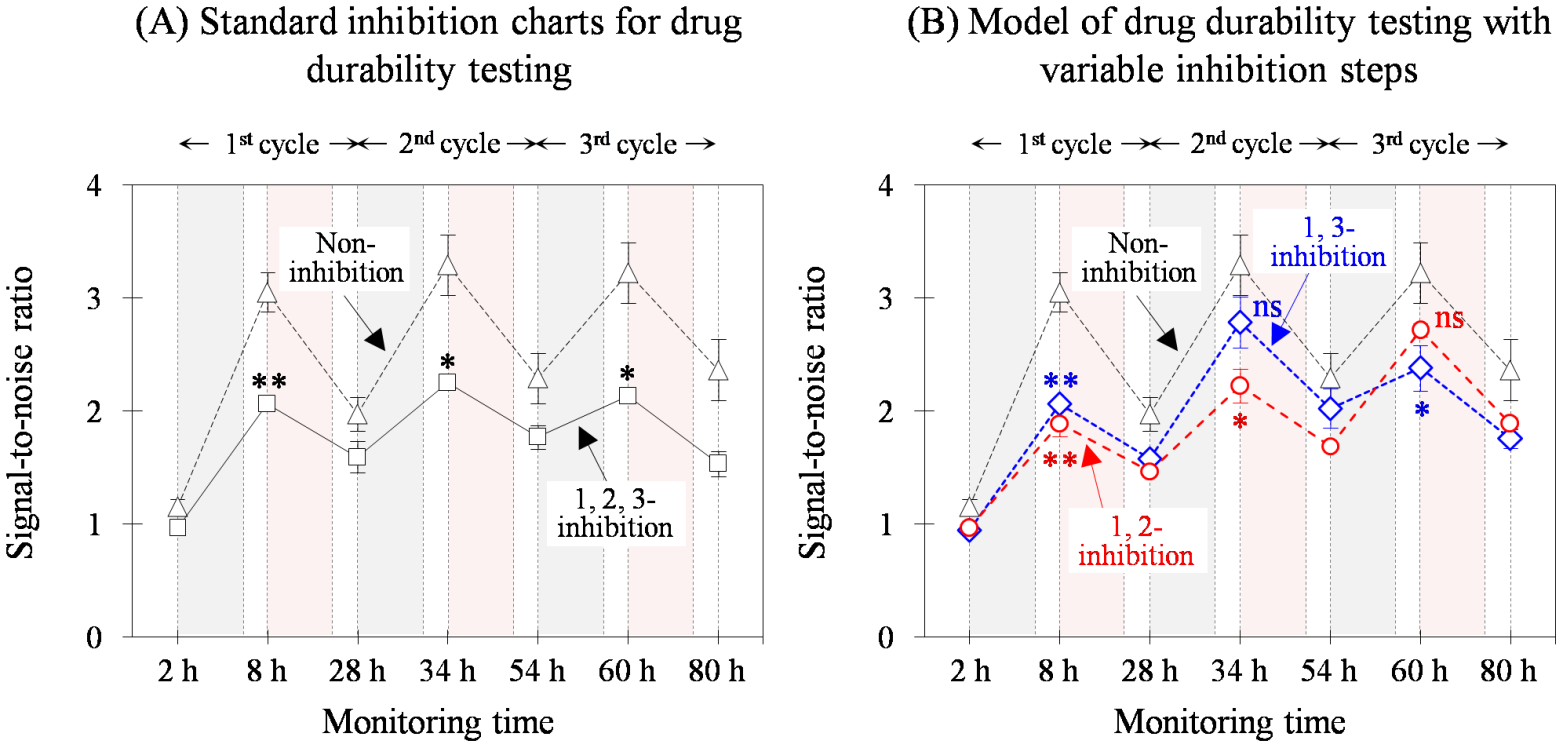
Simulation of the drug effect persistency testing using sodium salicylate. Pre-incubation with sodium salicylate during each cycle caused consistent anti-inflammatory responses to the three repeated bacterial stimulations (“1,2,3-inhibition” as a positive control) compared to that without inhibition (Non-inhibition as negative control). However, when the treatment was skipped at either the second (“1,3-inhibition”) or third cycle (“1,2-inhibition”), the inhibitory effect was not shown at the corresponding stage. This may indicate that drug efficacy persisted for 1 day. Each measurement was repeated twice under the same conditions and the significance was indicated.

Another notable feature of the semi-continuous monitoring system is providing information about duration of the test drug effect. Sodium salicylate was used as inhibitor of NF-κB activation as in the routine test but we omitted it after the first cycle (Figure 6). Two additional experiments were simultaneously executed without chemical treatments as a negative control (Figure 6A, Non-inhibition) and with the addition of the test substances during all three cycles as a positive control (Figure 6A, “1,2,3-inhibition”). When the cell culture was not chemically treated in the second cycle, TLR density increased compared to that of the controls, showing no significance of inhibition (Figure 6B, “1,3-inhibition”). When the chemical treatment was not employed in the third cycle, a similar response was obtained only at that stage (Figure 6B, “1,2-inhibition”). Nevertheless, increased TLR levels remained within the density boundaries of the two controls, revealing that such irregular inhibitor treatment did not irreversibly disturb cellular physiology.

As one of the stimulation-and-restoration cycles was not treated with sodium salicylate during the efficacy persistence testing, the substance used in the preceding culture was mostly washed out and the remainder underwent hydrolysis [41]. Such a removal process may limit the effective duration, although the Iκβ kinase binding complex slowly dissociated. The procedures could match with those under *in vivo* conditions. When a high dose of aspirin is ingested (e.g., >4 g), the half-life is 15 to 30 h [48]. This results from saturation of the biotransformation pathways concerned with decomposition to salicylate acid and salicyl phenolic glucuronide [49]. Renal excretion of salicylic acid becomes increasingly important as the metabolic pathways become saturated, which could be equivalent to the washing procedure under cell culture conditions. Thus, the duration of the effect estimated by *in vitro* cell culture can furnish us useful information comparable to that obtained via *in vivo* metabolic pathways.

The TLR-based biosensing approach could be unique in the analytical aspect showing the human cellular responses in semi-continuous manner. Other conventional methods, for example, TLR mRNA detection and NF-κB translocation assay, required cell disruption, not allowing us to repeatedly analyze the immune system activation as mentioned earlier. In the case of cytokine [e.g., tumor necrosis factor-α (TNF-α)] assay, the cells may not be destroyed and, however, the concentration (<10 pg/mL) secreted from the human cell line was too low to be assayed using a commercial enzyme-linked immunosorbent assay kit [50]. Human cells are needed to investigate human drugs [14]. In addition, certain proteases such as matrix metalloproteinase-9 (MMP-9) are also induced to secrete by the inflammatory stimulation [51]. MMP-9 has been reported to release by cytokines (e.g., TNF-α, IL-1), which may direct our attention to its utilization as a next-generation biomarker for vascular and inflammatory diseases as well as for cancers [51]. It was detected by immunoassay based on fluorometry or label-free method [52]–[54].

Alternatively, a murine cell line such as RAW264.7 may be used to secrete cytokines in a level sufficient for immunological detection [50] and, thus, TNF-α and interleukin-6 (IL-6) were tested as markers for monitoring cellular responses (Figure S7). According to the pre-determined scheme (refer to Figure 3A), the cells underwent three consecutive stimulation-restoration cycles and the TNF-α level was then monitored as an indication of the responses to each cycle. Although the signal was high relative to the background in the initial response, the ratio decreased significantly as the same cells were exposed to the next repetitive bacterial stimulations. In contrast, the signal-to-noise ratio for IL-6 was much lower than that for TNF-α, but the initial value was reproduced in the next two cycles, which was also supported by a previous result [50]. Such a cytokine-based model may only be preferred in microbe surveillance for environmental monitoring and biodefense.

## Conclusions

The differential TLR biosensing concept proposed by combining TLR regulation switching with semi-continuous monitoring enabled us to provide highly informative data essential for screening anti-inflammatory drug candidates. The information could be used to estimate inhibition efficacy for TLR expression against repeated bacterial stimulations, cytotoxicity, and duration of the effect. These factors are required of candidates to pass to the next steps of the drug screening process prior to pre-clinical tests [14]. The efficacy of therapeutic drugs in human clinical trials can be faithfully predicted neither by a single cell culture nor *in vivo* animal model [55]. However, cell lines dividing actively *in vitro* offers relatively to animal models the convenience of preparation and culture, if the desired cell type is available. The cell line can be replaced under optimized testing conditions with primary cells extracted from human directly, with the advantages of having numerous available cell types and the similarity function of *in vivo* cells [56]. Therefore, our novel biosensing model based on mammalian cell culture may be useful as an alternative to the current model, in which animals are used for drug toxicity testing. We plan to further investigate the utility of the model to enhance the drug effects by formulating of mixed candidate substances or shifting the treatment of each cycle, which may be difficult to apply even to animals.

## Supporting Information

Figure S1 **Expressions of TLR1, TLR2, and TLR4 on different host cells stimulated with various bacterial lysate at a concentration of 0.001 mg/mL proteins.** *P. aeruginosa* and *V. haemolyticus* only induced the expression of TLR1 when compared with each background (None) for all cell lines. In contrast, *S. sonnei* stimulated all tested TLRs expression in the order of TLR4>TLR2>TLR1. Data are shown in mean ± SD (n = 4) and comparisons to the control (i.e., expression levels of non-stimulated cells) are marked as *** very highly significant (P<0.001), ** highly significant (P<0.01), or * significant (P<0.05). No significance (ns) was indicated otherwise.
(TIF)

Figure S2 **Supporting data for the inhibition of bacterial stimulus inflammation by sodium salisylate at the molecular level.** When the bacterial lysate of *P. aeruginosa* was added into the A549 cell culture, the mRNA level for bradykinin 2 receptor (B2R) was increased comparing to that of non-stimulated control (A, 0 mM). Such level, however, was shown to suppress in the presence of sodium salicylate, of which degree was proportional to the dose added. On the other hand, these results were not reproduced by the addition of acetaminophen, revealing no anti-inflammatory effect (B). The identical procedures were repeated four times, respectively.
(TIF)

Figure S3 **Construction of semi-continuous biosensing system and its utilization for monitoring of the TLR level on mammalian cells.** A chemiluminometric immuno-sensing device was constructed by installing a cooled CCD within a dark chamber (A). The stimulation-and-restoration cycle for the same cell culture was semi-continuously monitored by measuring the TLR level via immunoassay as the cellular response to repeated stimulations (B). These were then compared with those of chemical treatment with sodium salicylate in different modes (C and D; refer to the manuscript for details). All experiment was carried out in duplicate.
(TIF)

Figure S4 **Inhibition model for inflammation using TLR4 response to** ***S. sonnei*** **on A549.** The TLR4 expression was observed in response to two cyclic repeated bacterial stimulations (No treatment). Sodium salicylate (50 mM) was then used by sequential incubations with the stimulus agent (Pre-incubation), revealing inhibition of the TLR response to serial stimulations. Each experiment was repeated twice.
(TIF)

Figure S5 **Simulation of cytotoxicity testing by using excess CAPE.** When CAPE was used in the cell culture in a concentration equal to or higher than 180 µM, the cells were observed to be damaged (refer to Figure S6C for microscopic observation of the culture). This resulted in high signals from both of the stimulated and non- stimulated cultures comparing to those without treatment (compares data in (A) and (B) at 28 h at the end of the first cycle). Such effect caused a signal-to-noise ratio deviated from a pattern parallel to the control without inhibition. The identical experiments were carried out in duplicate, respectively.
(TIF)

Figure S6 **Microscopic observation of the cell cultures treated with different doses of CAPE.** When relatively low doses of CAPE were added (e.g., <100 µM), the cells grown on the solid surfaces were unaffectedly healthy at the end of the first cycle at 28 h (A and B). However, the substance exceeding a certain concentration (e.g., 180 µM) caused cell detachment and the washout from the culture after medium change (C).
(TIF)

Figure S7 **Biosensing of cytokines for cellular response to bacterial stimulation on a murine cell line, RAW264.7.** Cyclic responses of the cells to repetitive stimulations were then monitored by measuring two cytokines, TNF-α and IL-6, at the same time based on a modified scheme of stimulation-restoration processes: 2 h-starvation, 2 h- stimulation, 2 h-restoration, and additional 20 h-restoration. The TNF-α level was kept decreasing as the stimulation was repeated in the three cycles while the IL-6 concentration was maintained about constant. The experiments were repeated twice under each condition.
(TIF)

Text S1 **Supplementary information for the experimental materials and methods mentioned in the main manuscript.**
(DOCX)

## References

[1] Ferrero-Miliani L, Nielsen OH, Andersen PS, Girardin SE (2007) Chronic inflammation: importance of NOD2 and NALP3 in interleukin-1beta generation. Clin Exp Immunol 147: 227–235. 17223962 10.1111/j.1365-2249.2006.03261.x PMC1810472

[2] Weiss U (2008) Inflammation. Nature 454: 427. 18650912 10.1038/454427a

[3] Nathan C (2002) Points of control in inflammation. Nature 420: 846–852. 12490957 10.1038/nature01320

[4] Ghaemmaghami AM, Hancock MJ, Harrington H, Kaji H, Khademhosseini A (2012) Biomimetic tissues on a chip for drug discovery. Drug Discov Today 17: 173–181. 22094245 10.1016/j.drudis.2011.10.029 PMC3273552

[5] Snyder DS (1976) Effect of topical indomethacin on UVR-induced redness and prostaglandin E levels in sunburned guinea pig skin. Prostaglandins 11: 631–643. 959567 10.1016/0090-6980(76)90066-6

[6] Intahphuak S, Panthong A, Kanjanapothi D, Taesotikul T, Krachangchaeng C, et al (2004) Anti-inflammatory and analgesic activities of Mallotus spodocarpus Airy shaw. J Ethnopharmacol 90: 69–72. 14698511 10.1016/j.jep.2003.09.026

[7] Xu Z, Zhou J, Cai J, Zhu Z, Sun X, et al (2012) Anti-inflammation effects of hydrogen saline in LPS activated macrophages and carrageenan induced paw oedema. J Inflamm (Lond) 9: 2. 22296736 10.1186/1476-9255-9-2 PMC3298497

[8] Lan T, Kandimalla ER, Yu D, Bhagat L, Li Y, et al (2007) Stabilized immune modulatory RNA compounds as agonists of Toll-like receptors 7 and 8. Proc Natl Acad Sci U S A 104: 13750–13755. 17698957 10.1073/pnas.0706059104 PMC1959454

[9] Panter G, Kuznik A, Jerala R (2009) Therapeutic applications of nucleic acids as ligands for Toll-like receptors. Curr Opin Mol Ther 11: 133–145. 19330719

[10] Singh AK, Parashar A, Singh AK, Singh R (2013) Pre-natal/juvenile chlorpyrifos exposure associated with immunotoxicity in adulthood in Swiss albino mice. J Immunotoxicol 10: 141–149. 23078415 10.3109/1547691X.2012.700653

[11] Hennessy EJ, Parker AE, O'Neill LA (2010) Targeting Toll-like receptors: emerging therapeutics? Nat Rev Drug Discov 9: 293–307. 20380038 10.1038/nrd3203

[12] Ehlers M, Ravetch JV (2007) Opposing effects of Toll-like receptor stimulation induce autoimmunity or tolerance. Trends Immunol 28: 74–79. 17197239 10.1016/j.it.2006.12.006

[13] Meneghin A, Hogaboam CM (2007) Infectious disease, the innate immune response, and fibrosis. J Clin Invest 117: 530–538. 17332880 10.1172/JCI30595 PMC1804377

[14] Huh D, Matthews BD, Mammoto A, Montoya-Zavala M, Hsin HY, et al (2010) Reconstituting organ-level lung functions on a chip. Science 328: 1662–1668. 20576885 10.1126/science.1188302 PMC8335790

[15] Leung MC, Williams PL, Benedetto A, Au C, Helmcke KJ, et al (2008) Caenorhabditis elegans: an emerging model in biomedical and environmental toxicology. Toxicol Sci 106: 5–28. 18566021 10.1093/toxsci/kfn121 PMC2563142

[16] Irazoqui JE, Troemel ER, Feinbaum RL, Luhachack LG, Cezairliyan BO, et al (2010) Distinct pathogenesis and host responses during infection of C. elegans by P. aeruginosa and S. aureus. PLoS Pathog 6: e1000982. 20617181 10.1371/journal.ppat.1000982 PMC2895663

[17] Gosai SJ, Kwak JH, Luke CJ, Long OS, King DE, et al (2010) Automated high-content live animal drug screening using C. elegans expressing the aggregation prone serpin alpha1-antitrypsin Z. PLoS One. 5: e15460. 10.1371/journal.pone.0015460 PMC2980495 21103396

[18] Medzhitov R (2001) Toll-like receptors and innate immunity. Nat Rev Immunol 1: 135–145. 11905821 10.1038/35100529

[19] Ospelt C, Gay S (2010) TLRs and chronic inflammation. Int J Biochem Cell Biol 42: 495–505. 19840864 10.1016/j.biocel.2009.10.010

[20] Kaplan AP, Joseph K, Silverberg M (2002) Pathways for bradykinin formation and inflammatory disease. J Allergy Clin Immunol 109: 195–209. 11842287 10.1067/mai.2002.121316

[21] Margolius HS (1995) Theodore Cooper Memorial Lecture. Kallikreins and kinins. Some unanswered questions about system characteristics and roles in human disease. Hypertension 26: 221–229. 7635529 10.1161/01.hyp.26.2.221

[22] Kopp E, Ghosh S (1994) Inhibition of NF-kappa B by sodium salicylate and aspirin. Science 265: 956–959. 8052854 10.1126/science.8052854

[23] Seabrook GR, Bowery BJ, Heavens R, Brown N, Ford H, et al (1997) Expression of B1 and B2 bradykinin receptor mRNA and their functional roles in sympathetic ganglia and sensory dorsal root ganglia neurones from wild-type and B2 receptor knockout mice. Neuropharmacology 36: 1009–1017. 9257945 10.1016/s0028-3908(97)00065-8

[24] Sabourin T, Morissette G, Bouthillier J, Levesque L, Marceau F (2002) Expression of kinin B(1) receptor in fresh or cultured rabbit aortic smooth muscle: role of NF-kappa B. Am J Physiol Heart Circ Physiol. 283: H227–237. 10.1152/ajpheart.00978.2001 12063295

[25] Raymond P, Drapeau G, Raut R, Audet R, Marceau F, et al (1995) Quantification of des-Arg9-bradykinin using a chemiluminescence enzyme immunoassay: application to its kinetic profile during plasma activation. J Immunol Methods 180: 247–257. 7714339 10.1016/0022-1759(94)00320-v

[26] Cugno M, Agostoni P, Brunner HR, Gardinali M, Agostoni A, et al (2000) Plasma bradykinin levels in human chronic congestive heart failure. Clin Sci (Lond) 99: 461–466. 11052927

[27] Granstrom E, Hamberg M, Hansson G, Kindahl H (1980) Chemical instability of 15-keto-13,14-dihydro-PGE2: the reason for low assay reliability. Prostaglandins 19: 933–957. 7384561 10.1016/0090-6980(80)90127-6

[28] Fitzpatrick FA, Aguirre R, Pike JE, Lincoln FH (1980) The stability of 13,14-dihydro-15 keto-PGE2. Prostaglandins 19: 917–931. 7384560 10.1016/0090-6980(80)90126-4

[29] Stamme C, Walsh E, Wright JR (2000) Surfactant protein A differentially regulates IFN-gamma- and LPS-induced nitrite production by rat alveolar macrophages. Am J Respir Cell Mol Biol 23: 772–779. 11104730 10.1165/ajrcmb.23.6.4083

[30] Scheinman RI, Cogswell PC, Lofquist AK, Baldwin AS Jr (1995) Role of transcriptional activation of I kappa B alpha in mediation of immunosuppression by glucocorticoids. Science 270: 283–286. 7569975 10.1126/science.270.5234.283

[31] Prado GN, Taylor L, Zhou X, Ricupero D, Mierke DF, et al (2002) Mechanisms regulating the expression, self-maintenance, and signaling-function of the bradykinin B2 and B1 receptors. J Cell Physiol 193: 275–286. 12384980 10.1002/jcp.10175

[32] Verstrepen L, Bekaert T, Chau TL, Tavernier J, Chariot A, et al (2008) TLR-4, IL-1R and TNF-R signaling to NF-kappaB: variations on a common theme. Cell Mol Life Sci 65: 2964–2978. 18535784 10.1007/s00018-008-8064-8 PMC11131687

[33] Collins T, Read MA, Neish AS, Whitley MZ, Thanos D, et al (1995) Transcriptional regulation of endothelial cell adhesion molecules: NF-kappa B and cytokine-inducible enhancers. Faseb J 9: 899–909. 7542214

[34] Cho IH, Jeon JW, Paek SH, Kim DH, Shin HS, et al (2012) Toll-like receptor-based immuno-analysis of pathogenic microorganisms. Anal Chem 84: 9713–9720. 23101931 10.1021/ac300668y

[35] Jeon JW, Cho IH, Ha UH, Seo SK, Paek SH (2014) Chemiluminometric Immuno-Analysis of Innate Immune Response against Repetitive Bacterial Stimulations for the Same Mammalian Cells. SCI REP-UK: Accepted. 10.1038/srep06011 PMC4127502 25109895

[36] Yin MJ, Yamamoto Y, Gaynor RB (1998) The anti-inflammatory agents aspirin and salicylate inhibit the activity of I(kappa)B kinase-beta. Nature 396: 77–80. 9817203 10.1038/23948

[37] Hertzberg RP, Pope AJ (2000) High-throughput screening: new technology for the 21st century. Curr Opin Chem Biol 4: 445–451. 10959774 10.1016/s1367-5931(00)00110-1

[38] Shin HS, Ha UH (2011) Up-regulation of bradykinin B2 receptor by Pseudomonas aeruginosa via the NF-kappaB pathway. Curr Microbiol 63: 138–144. 21626144 10.1007/s00284-011-9959-4

[39] Giard DJ, Aaronson SA, Todaro GJ, Arnstein P, Kersey JH, et al (1973) In vitro cultivation of human tumors: establishment of cell lines derived from a series of solid tumors. J Natl Cancer Inst 51: 1417–1423. 4357758 10.1093/jnci/51.5.1417

[40] Shin HS, Lee JH, Paek SH, Jung YW, Ha UH (2013) Pseudomonas aeruginosa-dependent upregulation of TLR2 influences host responses to a secondary Staphylococcus aureus infection. Pathog Dis 69: 149–156. 23913551 10.1111/2049-632X.12074

[41] Vane JR (1971) Inhibition of prostaglandin synthesis as a mechanism of action for aspirin-like drugs. Nat New Biol 231: 232–235. 5284360 10.1038/newbio231232a0

[42] Bellosillo B, Pique M, Barragan M, Castano E, Villamor N, et al (1998) Aspirin and salicylate induce apoptosis and activation of caspases in B-cell chronic lymphocytic leukemia cells. Blood 92: 1406–1414. 9694730

[43] Mousavi SA, Malerod L, Berg T, Kjeken R (2004) Clathrin-dependent endocytosis. Biochem J 377: 1–16. 14505490 10.1042/BJ20031000 PMC1223844

[44] Natarajan K, Singh S, Burke TR Jr, Grunberger D, Aggarwal BB (1996) Caffeic acid phenethyl ester is a potent and specific inhibitor of activation of nuclear transcription factor NF-kappa B. Proc Natl Acad Sci U S A. 93: 9090–9095. 10.1073/pnas.93.17.9090 PMC38600 8799159

[45] Catella-Lawson F, Reilly MP, Kapoor SC, Cucchiara AJ, DeMarco S, et al (2001) Cyclooxygenase inhibitors and the antiplatelet effects of aspirin. N Engl J Med 345: 1809–1817. 11752357 10.1056/NEJMoa003199

[46] Hinz B, Cheremina O, Brune K (2008) Acetaminophen (paracetamol) is a selective cyclooxygenase-2 inhibitor in man. FASEB J 22: 383–390. 17884974 10.1096/fj.07-8506com

[47] Tian H, Ip L, Luo H, Chang DC, Luo KQ (2007) A high throughput drug screen based on fluorescence resonance energy transfer (FRET) for anticancer activity of compounds from herbal medicine. Br J Pharmacol 150: 321–334. 17179946 10.1038/sj.bjp.0706988 PMC2013898

[48] Chyka PA, Erdman AR, Christianson G, Wax PM, Booze LL, et al (2007) Salicylate poisoning: an evidence-based consensus guideline for out-of-hospital management. Clin Toxicol (Phila) 45: 95–131. 17364628 10.1080/15563650600907140

[49] Prescott LF, Balali-Mood M, Critchley JA, Johnstone AF, Proudfoot AT (1982) Diuresis or urinary alkalinisation for salicylate poisoning? Br Med J (Clin Res Ed) 285: 1383–1386. 10.1136/bmj.285.6352.1383 PMC1500395 6291695

[50] Huttunen K, Hyvarinen A, Nevalainen A, Komulainen H, Hirvonen MR (2003) Production of proinflammatory mediators by indoor air bacteria and fungal spores in mouse and human cell lines. Environ Health Perspect 111: 85–92. 12515684 10.1289/ehp.5478 PMC1241310

[51] Vandooren J, Van den Steen PE, Opdenakker G (2013) Biochemistry and molecular biology of gelatinase B or matrix metalloproteinase-9 (MMP-9): the next decade. Crit Rev Biochem Mol Biol 48: 222–272. 23547785 10.3109/10409238.2013.770819

[52] Stryer L (1978) Fluorescence energy transfer as a spectroscopic ruler. Annu Rev Biochem 47: 819–846. 354506 10.1146/annurev.bi.47.070178.004131

[53] Vazquez de Lara LG, Umstead TM, Davis SE, Phelps DS (2003) Surfactant protein A increases matrix metalloproteinase-9 production by THP-1 cells. Am J Physiol Lung Cell Mol Physiol 285: L899–906. 12842807 10.1152/ajplung.00082.2003

[54] Wu SH, Lee KL, Chiou A, Cheng X, Wei PK (2013) Optofluidic platform for real-time monitoring of live cell secretory activities using Fano resonance in gold nanoslits. Small 9: 3532–3540. 23606668 10.1002/smll.201203125

[55] HogenEsch H, Nikitin AY (2012) Challenges in pre-clinical testing of anti-cancer drugs in cell culture and in animal models. J Control Release 164: 183–186. 22446384 10.1016/j.jconrel.2012.02.031 PMC3387503

[56] Liu Q, Wu C, Cai H, Hu N, Zhou J, et al.. (2014) Cell-Based Biosensors and Their Application in Biomedicine. Chem Rev. 10.1021/cr2003129 24905074

