# Supplementary figures and images for: In Vitro Inflammation Inhibition Model Based on Semi-Continuous Toll-Like Receptor Biosensing
Source: PLoS One. 2014 Aug 19;9(8):e105212. doi: 10.1371/journal.pone.0105212 (PMC4138127; doi:10.1371/journal.pone.0105212)

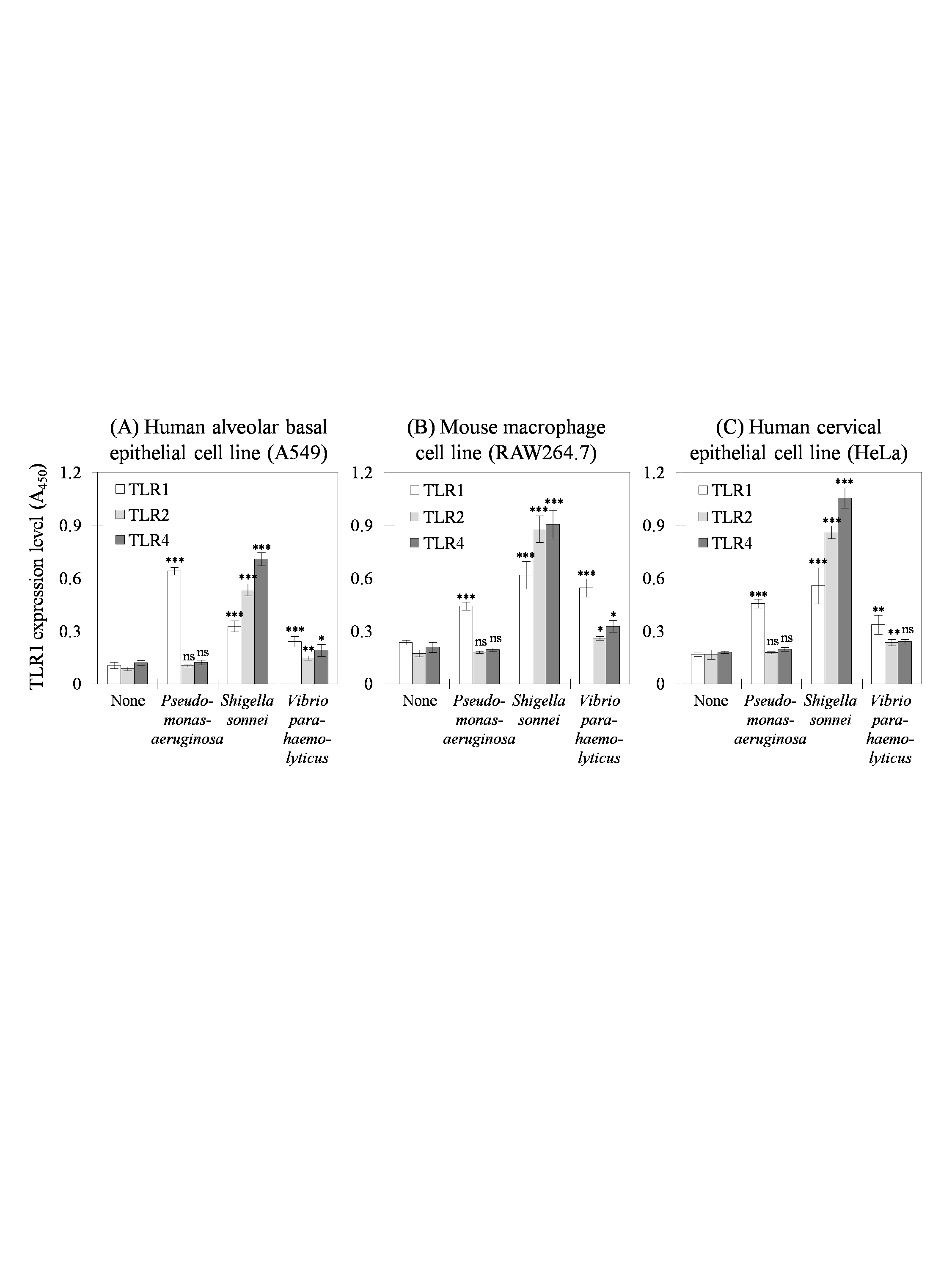

Supplement: Figure S1 — Expressions of TLR1, TLR2, and TLR4 on different host cells stimulated with various bacterial lysate at a concentration of 0.001 mg/mL proteins. P. aeruginosa and V. haemolyticus only induced the expression of TLR1 when compared with each background (None) for all cell lines. In contrast, S. sonnei stimulated all tested TLRs expression in the order of TLR4>TLR2>TLR1. Data are shown in mean ± SD (n = 4) and comparisons to the control (i.e., expression levels of non-stimulated cells) are marked as *** very highly significant (P<0.001), ** highly significant (P<0.01), or * significant (P<0.05). No significance (ns) was indicated otherwise. (TIF) [file pone.0105212.s001.tif]

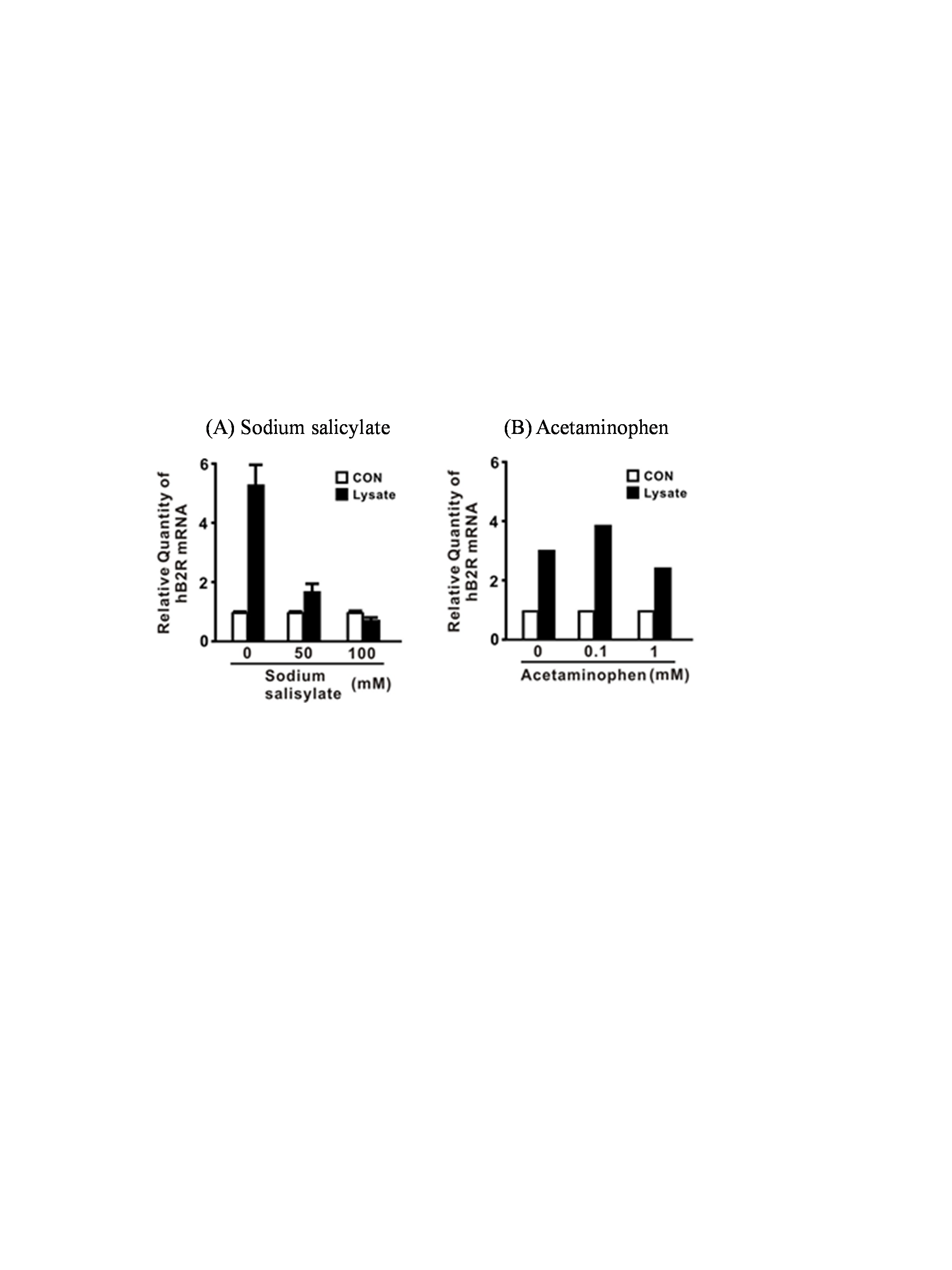

Supplement: Figure S2 — Supporting data for the inhibition of bacterial stimulus inflammation by sodium salisylate at the molecular level. When the bacterial lysate of P. aeruginosa was added into the A549 cell culture, the mRNA level for bradykinin 2 receptor (B2R) was increased comparing to that of non-stimulated control (A, 0 mM). Such level, however, was shown to suppress in the presence of sodium salicylate, of which degree was proportional to the dose added. On the other hand, these results were not reproduced by the addition of acetaminophen, revealing no anti-inflammatory effect (B). The identical procedures were repeated four times, respectively. (TIF) [file pone.0105212.s002.tif]

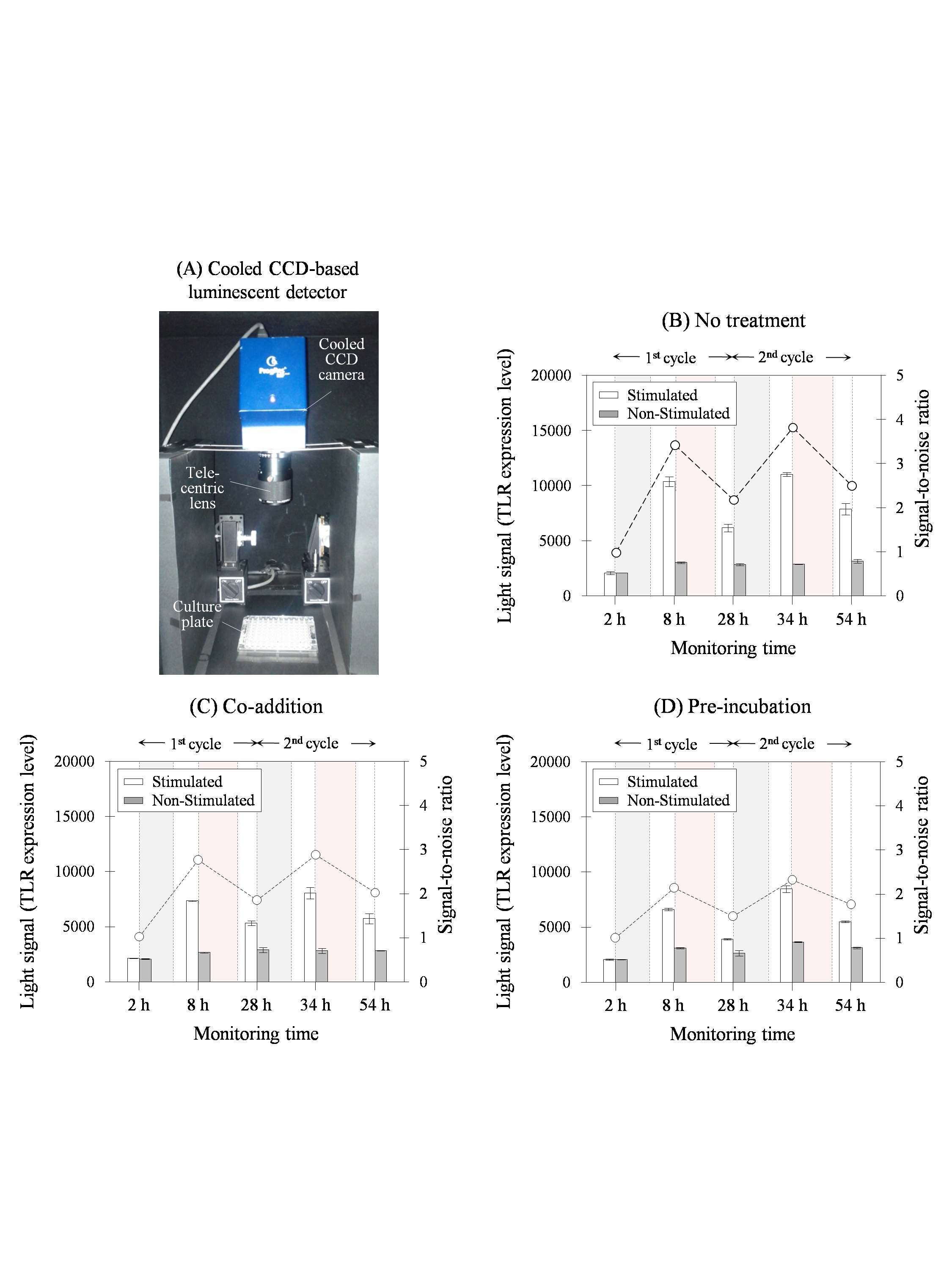

Supplement: Figure S3 — Construction of semi-continuous biosensing system and its utilization for monitoring of the TLR level on mammalian cells. A chemiluminometric immuno-sensing device was constructed by installing a cooled CCD within a dark chamber (A). The stimulation-and-restoration cycle for the same cell culture was semi-continuously monitored by measuring the TLR level via immunoassay as the cellular response to repeated stimulations (B). These were then compared with those of chemical treatment with sodium salicylate in different modes (C and D; refer to the manuscript for details). All experiment was carried out in duplicate. (TIF) [file pone.0105212.s003.tif]

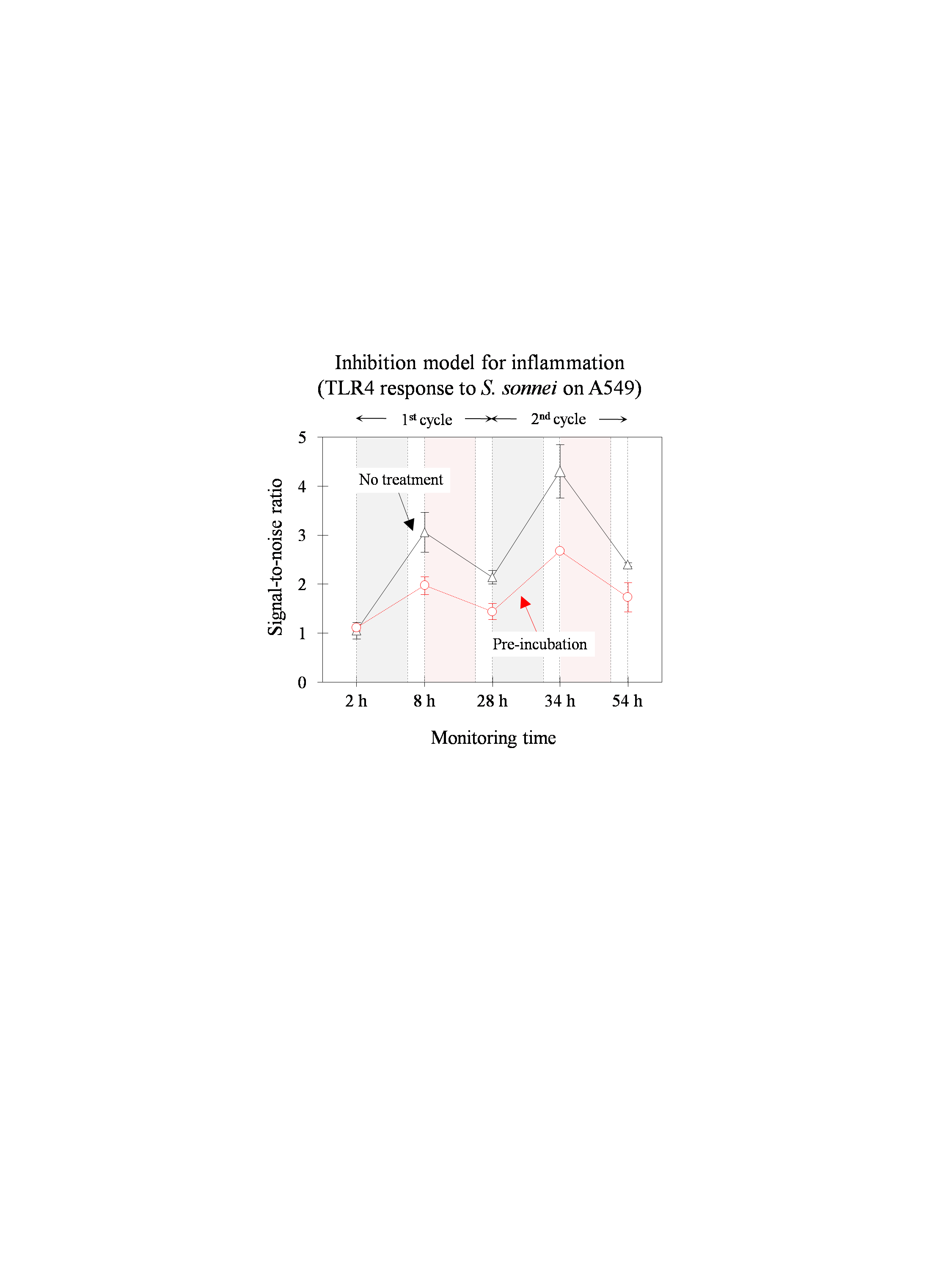

Supplement: Figure S4 — Inhibition model for inflammation using TLR4 response to S. sonnei on A549. The TLR4 expression was observed in response to two cyclic repeated bacterial stimulations (No treatment). Sodium salicylate (50 mM) was then used by sequential incubations with the stimulus agent (Pre-incubation), revealing inhibition of the TLR response to serial stimulations. Each experiment was repeated twice. (TIF) [file pone.0105212.s004.tif]

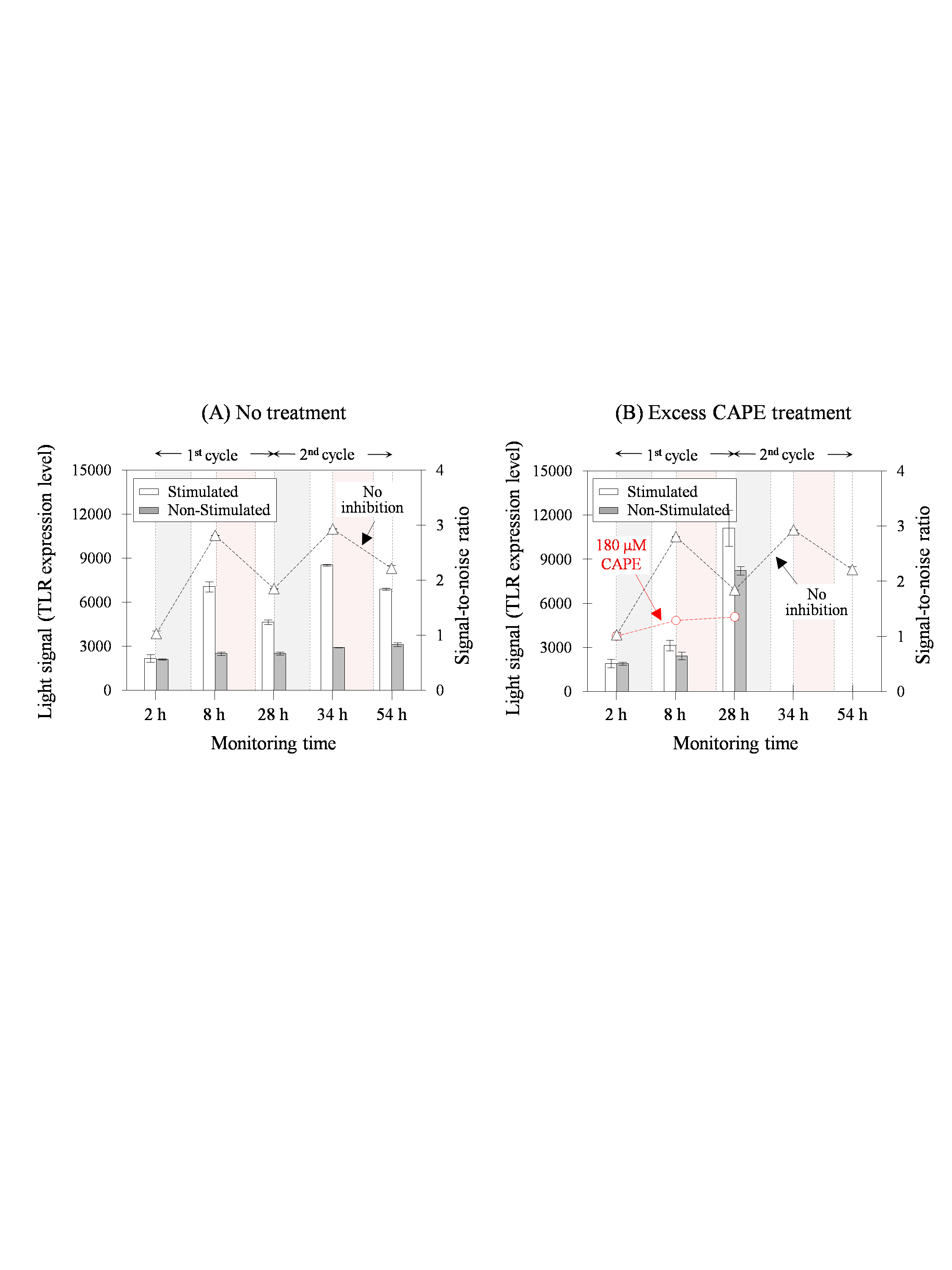

Supplement: Figure S5 — Simulation of cytotoxicity testing by using excess CAPE. When CAPE was used in the cell culture in a concentration equal to or higher than 180 µM, the cells were observed to be damaged (refer to Figure S6C for microscopic observation of the culture). This resulted in high signals from both of the stimulated and non- stimulated cultures comparing to those without treatment (compares data in (A) and (B) at 28 h at the end of the first cycle). Such effect caused a signal-to-noise ratio deviated from a pattern parallel to the control without inhibition. The identical experiments were carried out in duplicate, respectively. (TIF) [file pone.0105212.s005.tif]

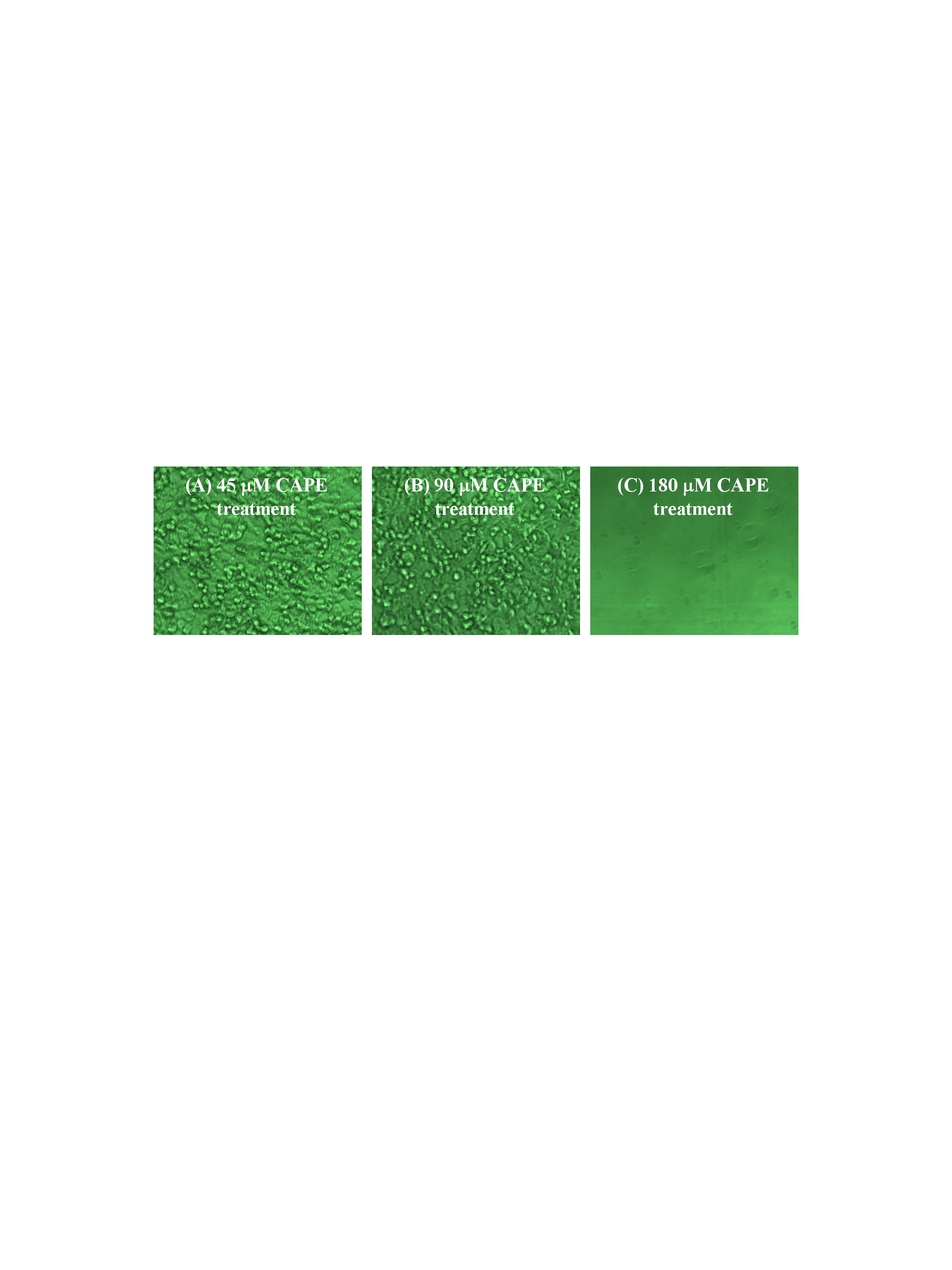

Supplement: Figure S6 — Microscopic observation of the cell cultures treated with different doses of CAPE. When relatively low doses of CAPE were added (e.g., <100 µM), the cells grown on the solid surfaces were unaffectedly healthy at the end of the first cycle at 28 h (A and B). However, the substance exceeding a certain concentration (e.g., 180 µM) caused cell detachment and the washout from the culture after medium change (C). (TIF) [file pone.0105212.s006.tif]

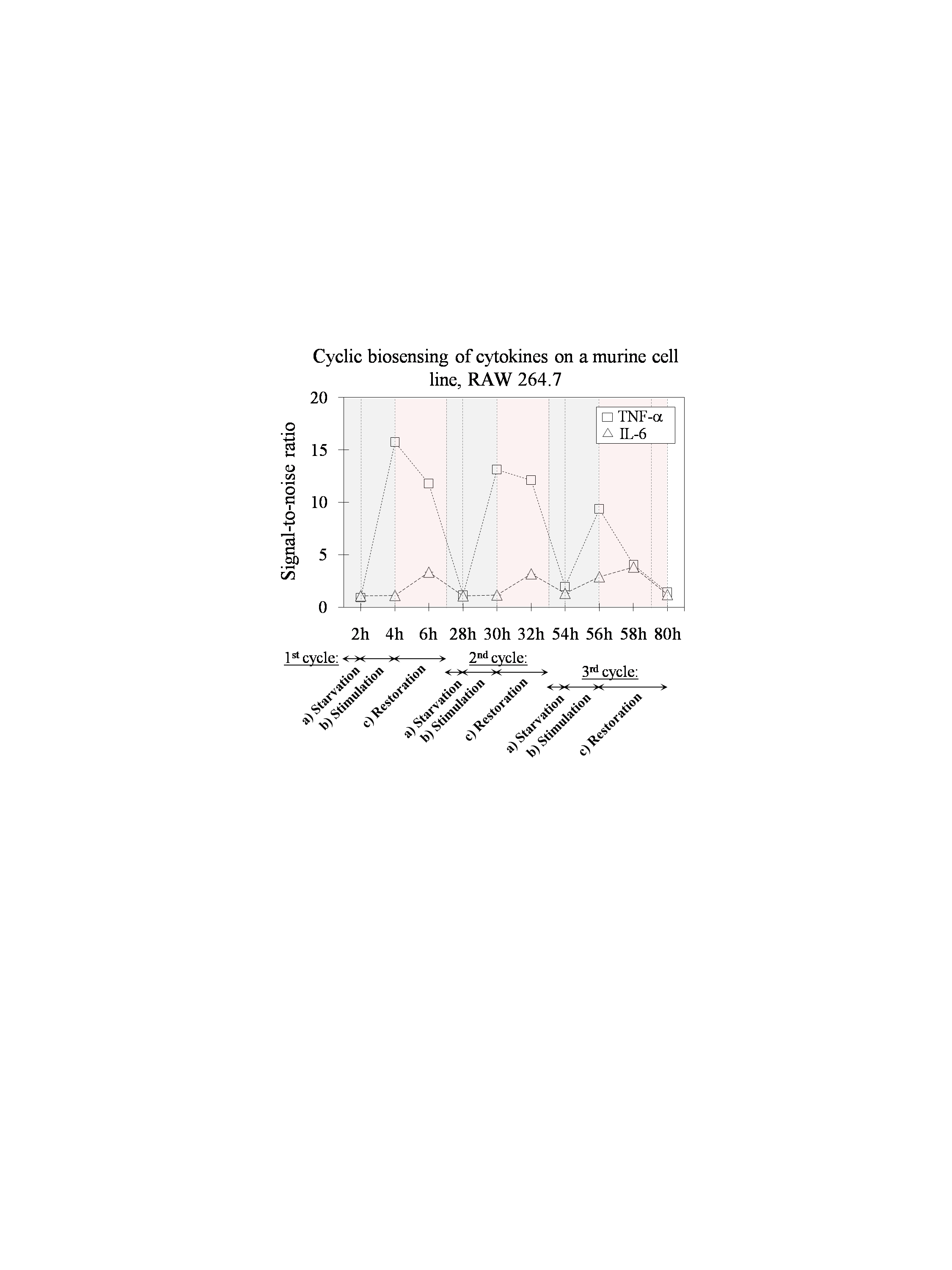

Supplement: Figure S7 — Biosensing of cytokines for cellular response to bacterial stimulation on a murine cell line, RAW264.7. Cyclic responses of the cells to repetitive stimulations were then monitored by measuring two cytokines, TNF-α and IL-6, at the same time based on a modified scheme of stimulation-restoration processes: 2 h-starvation, 2 h- stimulation, 2 h-restoration, and additional 20 h-restoration. The TNF-α level was kept decreasing as the stimulation was repeated in the three cycles while the IL-6 concentration was maintained about constant. The experiments were repeated twice under each condition. (TIF) [file pone.0105212.s007.tif]
