# Supplementary material for: In Vitro Inflammation Inhibition Model Based on Semi-Continuous Toll-Like Receptor Biosensing
Source: PLoS One. 2014 Aug 19;9(8):e105212. doi: 10.1371/journal.pone.0105212 (PMC4138127; doi:10.1371/journal.pone.0105212)
Supplement: Text S1 — Supplementary information for the experimental materials and methods mentioned in the main manuscript. (DOCX) [file pone.0105212.s008.docx]

**Supplementary Information**

**Materials and Methods**

**Materials**

Mammalian cell lines, A549 (ATCC CCL-185) and HeLa (ATCC CCL-2), were supplied by American Type Culture Collection (Manassas, VA), and RAW264.7 (KCLB 40071) was obtained from Korean Cell Line Bank (Seoul, South Korea). Dulbecco's Modified Eagle's Medium (DMEM) and Minimum Essential Medium Eagles with Earle's Balanced Salts (MEM/EBSS) were purchased from Welgene (Daegu, South Korea). Roswell Park Memorial Institute Medium (RPMI 1640), Dulbecco’s Phosphate Buffered Saline (DPBS(-/-)) modified with calcium and magnesium (DPBS(+/+)), fetal bovine serum (FBS), and penicillin-streptomycin solution were supplied by HyClone (Logan, UT). Goat anti-rabbit IgG coupled to horseradish peroxidase (HRP) and chemiluminescent substrate for HRP (Supersignal West Femto) were obtained from Thermo Fisher Scientific (Rockford, IL). Rabbit anti-TLR1 polyclonal antibody (H-90; 0.2 mg/mL), rabbit anti-TLR2 polyclonal antibody (H-175; 0.2 mg/mL), and rabbit anti-TLR4 polyclonal antibody (M-300; 0.2 mg/mL), caffeic acid phenethyl ester (CAPE), acetaminophen, and sodium salicylate were supplied by Santa Cruz Biotechnology (SantaCruz, CA). Casein (sodium salt type, extracted from milk), Tween-20, tryptone, yeast extract, sodium acetate, ethyl alcohol, Proclin 300, 3,3',5,5'-tetramethylbenzidine (TMB), and sodium chloride were purchased from Sigma (St. Louis, MO). SYBR Green PCR Master Mix, TaqMan Reverse Transcription Reagents, cytokine detection kits (Quantikine ELISA, TNF-α and IL-6), TRIzol Reagent, Trypsin-EDTA, and 96-well cell culture plate were obtained from KAPA Biosystems (Woburn, MA), R&D system (Minneapolis, MN), Applied Biosystems (Carlsbad, CA), Invitrogen (Carlsbad, CA), Gibco (Grand Island, NY), and Corning Incorporated (Corning, NY), respectively. Other reagents used in this study were of analytical grade.

**Preparation of Bacterial Lysate and Mammalian Cell**

**Preparation of bacterial lysate.** Bacterial lysates of *Pseudomonas aeruginosa* (PAK strain; *P. aeruginosa*), *Shigella sonnei* (*S. sonnei*), and *Vibrio parahaemolyticus* (*V. parahaemolyticus*) were prepared by the sonication method as described in a previous report[S[1](#_ENREF_1)]. Briefly, *P. aeruginosa* and *S. sonnei* were separately grown in Luria-Bertani broth at 37°C for 16 h, and *V. parahaemolyticus* was cultured in the medium containing 2% NaCl under the same conditions. The cells were then harvested by spinning down at 10,000 g at 4°C for 20 min. The cell pellets were suspended at a concentration of 1x10^8^ cells/mL in 10 mM phosphate buffer, pH 7.4, containing 140 mM NaCl (PBS), and the suspension was boiled for 10 min. The heat-killed cells were subsequently broken on ice for 2 min using a sonicator, which was then repeated 5 times with a 5 min interval. The residual cell debris was removed by centrifugation at 12,000 g at 4°C for 20 min. Each supernatant was measured for the protein concentration using Bradford assay[S[2](#_ENREF_2)], finally diluted to a 0.1 mg/mL protein concentration (corresponding to 1x10^7^ cells/mL for *P. aeruginosa*, 5x10^6^ cells/mL for *S. sonnei*, and 2x10^7^ cells/mL for *V. parahaemolyticus*) with PBS, and stored at -80°C after snap-frozen as aliquots.

**Cultivation of mammalian cell.** The maintenance of mammalian cells were basically conducted according to standard protocols[S[3](#_ENREF_3)]. The mammalian cell lines, A549, HeLa, and RAW264.7, were suspended in different culture media, RPMI1640, MEM/EBSS, and DMEM, supplemented with 10% FBS and 1% penicillin-streptomycin solution, respectively. The cell suspensions were separately dispensed into cell culture dishes (7 mL each), and cultivated in an incubator maintained at a 5% CO_2_ atmosphere and 37°C until the solid surfaces were 90% confluent. The medium was replaced with the fresh media every 2 days. To harvest the A549 cells, the culture medium was removed by suction, and the cells attached on the surfaces were washed with DPBS(-/-) 3 times. The trypsin-EDTA solution (1 mL) was added and incubated for up to 5 min until the cells were dispersed. The supplemented medium (20 mL) was added and the cell suspension was then transferred by gentle pipetting into three different dishes (7 mL each). For RAW264.7, the medium was exchanged with the fresh media (20 mL) and the cells were then harvested using a cell scrapper to detach them from the surfaces. After suspending the cells, they were divided into 5 dishes and the medium was adjusted to a constant volume. The subsequent cell expansion was carried out under the conditions identical to those described above. For cell stocking, the cells were diluted to 3x10^5^ cells/mL with each culture medium containing 5% FBS and 10% DMSO, and snap-frozen in liquid nitrogen and kept until used.

**Analytical Procedures for TLR Induced by Bacterial Stimulation**

**Pre-cultivation of mammalian cell for bacterial stimulation.** Prior to stimulation, the mammalian cell was cultivated by first suspending it in the culture medium (density: 3×10^5^ cells/mL) supplemented with 10% FBS and 1% penicillin-streptomycin solution. The cell suspension was then dispensed into 96-well culture plates (200 μL per well), and grown in an incubator maintained at a 5% CO_2_ atmosphere and 37°C for 12 h until the cells were stably attached to the bottom of the plate. After the medium was removed by suction, the wells were washed with DPBS (+/+) 3 times (200 μL per well). Serum-free medium was added into the wells and incubated under the same conditions for 2 h for starvation.

**Colorimetric immunoassay for TLRs without cell fixation.** Each bacterial lysate was diluted to 1/100 with the serum-free medium and added into the pre-cultivated mammalian cells after washing with DPBS (+/+) 3 times. After incubation under an environment of 5% CO_2_ atmosphere and 37°C for 2 h, the medium was removed by suction and each well was washed again with the culture medium containing 10% FBS 3 times. An antibody specific to the target TLR was diluted in the culture medium (1/150, 1/100, and 1/250) and incubated under the identical conditions for 1 h. After washing again under the same conditions, anti-rabbit IgG labeled with HRP diluted to 1/2500 was subsequently added into the wells and subsequently incubated as mentioned. After washing, a HRP substrate solution containing TMB (200 μL) was added and incubated for 15 min to generate the color signal. The reaction was stopped by adding 2 M sulfuric acid (50 μL) and the color signal was measured at an absorbance of 450 nm using a microtiter plate reader (VersaMax; Molecular Devices, Sunnyvale, CA). Each measured mean and standard deviation values were used to plot the receptor expression level with variation against the target variable by using the Excel program from Microsoft. Data are presented as mean ± standard deviation (SD). Statistical analyses were performed using one-way analysis of variance followed by Tukey’s post hoc multiple range tests[S[4](#_ENREF_4)] using the SPSS software package for Windows (SPSS Inc.; Chicago, IL, USA). A P < 0.05 was considered significant.

**Chemiluminescent immunoassay for TLRs.** To minimize cellular damage, a chemiluminometric substrate for HRP was invited to provide signal generation condition biocompatible with the animal cell culture. The TLRs expressed on the A549 cells by the bacterial stimulation were immunochemically analyzed as described earlier. The luminescent signal was produced by adding a substrate containing luminol and, after 3 min, measured by using a detector, which was devised by installing a cooled charge-coupled device (CCD) camera (ProgRes MF cool, JENOPTIK; Jena, Germany) within a dark chamber (50 x 50 x 50 cm). The camera was located on the top of the chamber and, when assembled, the signal was measured under dark conditions isolated from the ambient light. The CCD camera was initially adjusted to focus on the well bottom such that the image of the cells under cultivation was clearly captured. At the time of signal production, the luminescent signal was captured by using the CCD camera and then digitized by a Java-based image processing program, Image J (National Institutes of Health; Bethesda, MD). The optical densities were integrated and then used to plot the standard curve.

**Suppression of Cell Receptor Up-regulation by using Chemical Inhibitor**

**Inhibition of TLR expression at protein level.** By selecting sodium salicylate as inhibitor of NF-κB pathway, the inhibition conditions toward the concentration and treatment timing were first optimized via TLR-based colorimetric detection. The A549 cells were dispensed into 96-well culture plates and stably attached to the bottom of the plate as described. After washing, the cells were treated with the inhibitor in different manners, co-incubation with the bacterial lysate or their sequential incubation. For co-incubation, the cells were undergone starvation in serum-free medium for 2 h, and incubated in the medium containing stimulus agent and different doses of sodium salicylate for 2 h. After washing with the FBS-containing medium, the same immuno-analytical procedure as used for the TLR-based biosensing above was followed and the TLR1 level was then quantified by using the TMB-containing HRP substrate as mentioned earlier. For sequential incubation, the inhibitor was first added at the time of starvation and then stimulated with the lysate at the next step. The rest of the procedure was remained the same as indicated above. The identical protocol was repeated except for the absence of the inhibitor as the negative control, and all experiment was carried out in duplicate. The same protocol was applied to inhibition testing with CAPE and acetaminophen.

**Inhibition of BR expression at molecular level.** To verify anti-inflammation effect by sodium salicylate, the suppression of B2R expression was determined by measuring the mRNA level of the bacterially stimulated animal cells against the inhibitor concentration. In this case, sodium salicylate diluted in the serum-free medium was first incubated at the starvation stage as described earlier. The lysate was diluted 1/100 with the serum-free medium and then added into the pre-cultivated A549 cells after washing with DPBS (+/+) 3 times. The stimulated cells were incubated under an environment of 5% CO_2_ atmosphere and 37°C for 4 h. After washing again, total RNA was isolated from the stimulated cells using TRIzol Reagent according to the manufacturer’s instruction. Briefly, Trizol reagent (0.5 mL) was added into each culture and vigorously agitated for resuspension at room temperature for 10 min. The cell suspensions were separately transferred into each microtube containing chloroform (100 μL) and gently mixed under the same conditions. After spun down, each supernatant was transferred into a new microtube and combined with the same volume of 2-isopropanol (150 μL), and the mixture was incubated for 10 min. After spun down again, the precipitated total RNA was washed with 75% ethanol (800 μL), which was repeated 2 times. The excess ethanol was evaporated from the pellet containing total RNA at room temperature. The RNA was dissolved in diethylpyrocarbonate-treated water (50 μL) and the solution was incubated at 55 to 60°C for 10 min**.**

The extracted RNA was used to synthesize the complementary DNA (cDNA) by employing TaqMan Reverse Transcription Reagents according to the guide provided by the manufacturer. Reverse transcription was performed on MJ Mini Thermal Cycler (Bio-Rad, Hercules, CA) under the following thermal cyclic conditions: for annealing, 25°C for 10 min; for elongation, 37°C for 1 h and 42°C for 1 h; and for inactivation, 95°C for 5 min. After the produced cDNA was qualitatively checked on NanoDrop (Thermo Fisher Scientific Inc, Waltham, MA), it was quantified via real-time polymerase chain reaction (RT-PCR). Two primers for the human B2R gene were designed to have sequences of 5’-GGGCACACTGCGGACCT-3’ or 5’-GCGT TTGCTCACTGTCTGCTC-3’, respectively. To monitor extended DNA resulting from the RT-PCR, SYBR Green PCR Master Mix was added for intercalation by following the manufacturer’s instruction. RT-PCR was performed within ABI 7500 Real-Time PCR System (Applied Biosystems, Carlsbad, CA) using the following thermal cyclic conditions: for stage 1, 50°C for 2 min and then 95°C for 10 min; and for stage 2, 95°C for 15 second and then 60°C for 1 min. After the first thermal cycling at stage 1, stage 2 was repeated for 40 cycles. The same procedure was repeated with different primers (5’-CCCTCCAAAATCAAGTGG-3’ and 5’-CCATCCACAGTCTTCTGG-3’) for human glyceraldehyde 3-phosphate dehydrogenase (hGAPDH) gene as control. Relative quantities of B2R mRNA were calculated using the comparative threshold cycle method^S5^ and then normalized toward that for hGAPDH to determined the levels of mRNA used in each test.

**Semi-continuous Biosensing Model for Anti-inflammation**

**Semi-continuous response to repetitive bacterial stimulations.** The cellular response to repetitive bacterial stimulations was monitored by immunochemically measuring the TLR1 level expressed on the same A549 cells according to the up-and-down regulation switching. For the pre-cultivated cells, the background level of TLR1 was first measured via antigen-antibody bindings and subsequent luminescent signal generation as described above. Immediately after signal detection, the culture was washed with the medium containing 10% FBS 3 times such that the time elapsed from the substrate addition was less than 5 min. In the first cycle, the culture was starved in the serum-free medium for 2 h, stimulated with the bacterial lysate for 2 h, and the TLR1 density was determined by employing the same immunochemical procedure. The same culture was subsequently restored in the serum-containing medium for 18 h. For the next cycles, the identical procedure was repeated. The whole process was also negatively controlled without adding the stimulus agent, which was used to yield the signal-to-background ratio against time.

**Construction of inhibition model for inflammation.** To demonstrate the utility of the semi-continuous monitoring for repetitive stimulations, the same biosensing scheme based on TLR regulation switching was employed without cellular damage. As mentioned, sodium salicylate (50 mM) as inhibitor was incubated either with the bacterial lysate at the same time or by pre-addition at the starvation step. The cellular responses were cyclically monitored twice and the patterns were compared with that of the control obtained without treatment of the inhibitor.

**Simulation of anti-inflammatory substance screening.** For simulation of the screening procedure, CAPE and acetaminophen were selected as positive or negative candidate, respectively. Prior to testing, two standard curves were prepared by semi-continuously monitoring the cyclic TLR levels for inflammation against the repetitive bacterial stimulations and their inhibited level in the presence of sodium salicylate (50 mM), respectively. In this case, the inhibitor was sequentially incubated to enhance the anti-inflammation effect. Each of the whole process was also negatively controlled without adding the bacterial lysate such that the signal-to-background ratio can be calculated to finally plot the standard curves against time. The two candidates were then tested by serially adding each (90 μM for CAPE and 10 mM for acetaminophen) into the cell culture according to the scheme of semi-continuous biosensing as described.

**Estimation for anti-inflammatory duration.** To estimate drug effect persistency for sodium salicylate, the identical procedure for the inhibition testing was used except for omission of the chemical treatment in one of the two cycles after the addition in the first cycle. As described earlier, the two standard curves were first prepared and then used to compare with the results for the persistency testing.

**Measurement of Cytokines for Cellular Responses to Repetitive Bacterial Stimulations**

**Sampling from cell cultures under repetitive bacterial stimulations.** To monitor cellular responses to bacterial stimulations, the mammalian cells (RAW264.7) were cyclically stimulated with bacterial lysate according to stimulation-restoration process. The pre-cultivated cells (12 h) were washed with the medium containing 10% FBS 3 times. In the first cycle, the culture was starved in the serum-free medium for 2 h, and the culture sup was first collected and stored at 4°C for later analysis of the background level of cytokine. After rinsing with the medium containing 10% FBS 3 times, the diluted lysate was transferred and, after stimulation for 2 h, the sup was collected. After washing again, the medium containing 10% FBS was added and cultivated for 2 h, and the sup was finally collected for the cycle. The same cells were subsequently processed for restoration in the serum-containing medium for 20 h. For the second and third cycles, the same procedure was repeated to collect the culture sup samples each stage. The whole process was also negatively controlled without adding the stimulus agent.

**Immunoassays for pro-inflammatory cytokines.** Two cytokines, TNF-α and IL-6, produced from the mammalian cells during the stimulation-restorations processes were quantitatively measured by using the commercial ELISA kits. The collected samples were diluted (1/20 for TNF-α and 1/2 for IL-6) with each assay buffer provided in the kits. The diluted samples (100 μL) were transferred into the respective microtiter plate wells and reacted at room temperature for 2 h. The standard samples were also diluted with each assay buffer according to the guided protocol and handled using the same procedures. After rinsing the wells with the washing buffer 5 times, each detection antibody labeled with HRP was subsequently reacted. After washing again, the HRP substrate solution buffer (100 μL) was added into each well and incubated for 15 min. The reaction was stopped by adding the stop solution (100 μL) and the color signal was spectrophotometrically measured as described above.

**Supplementary References**

S1. Shin HS, Ha UH (2011) Up-regulation of bradykinin B2 receptor by Pseudomonas aeruginosa via the NF-kappaB pathway. Curr Microbiol 63: 138-144.

S2. Zor T, Selinger Z (1996) Linearization of the Bradford protein assay increases its sensitivity: theoretical and experimental studies. Anal Biochem 236: 302-308.

S3. Giard DJ, Aaronson SA, Todaro GJ, Arnstein P, Kersey JH, et al. (1973) In vitro cultivation of human tumors: establishment of cell lines derived from a series of solid tumors. J Natl Cancer Inst 51: 1417-1423.

S4. Shin HS, Lee JH, Paek SH, Jung YW, Ha UH (2013) Pseudomonas aeruginosa-dependent upregulation of TLR2 influences host responses to a secondary Staphylococcus aureus infection. Pathog Dis 69: 149-156.
